# Effect of minocycline, methyl prednisolone, or combination treatment on the colonic bacterial population in a state of colonic inflammation using the murine dextran sulfate sodium model

**DOI:** 10.1186/s12934-023-02242-8

**Published:** 2023-11-10

**Authors:** Maitham A. Khajah, Sanaa Hawai

**Affiliations:** College of Pharmacy, Kuwait University, PO Box 24923, 13110 Safat, Kuwait

**Keywords:** Colitis, DSS, Minocycline, Methyl prednisolone, Microbiota, Fecal 16s metagenomics

## Abstract

**Background:**

Several reports demonstrated anti-inflammatory properties of minocycline in various inflammatory disorders including colitis. We have experimental evidence suggesting synergistic anti-inflammatory effect of minocycline with methyl prednisolone in reducing colitis severity in mice, but if this effect is in part related to modulating the composition of colonic microbiota is still unknown.

**Methods:**

the effect of vehicle (V), minocycline (M), methyl prednisolone (MP), or combination (C) regimen on the composition of the microbiota of mice in a state of colon inflammation compared to untreated (UT) healthy mice was determined using 16s metagenomic sequencing, and the taxonomic and functional profiles were summarized.

**Results:**

Overall, the bacterial flora from the phylum *Firmicutes* followed by *Bacteroidota* were found to be predominant in all the samples. However, the composition of *Firmicutes* was decreased relatively in all the treatment groups compared to UT group. A relatively higher percentage of *Actinobacteriota* was observed in the samples from the C group. At the genus level, *Muribaculaceae*, *Bacteroides*, *Bifidobacterium*, and *Lactobacillus* were found to be predominant in the samples treated with both drugs (C). Whereas “*Lachnospiraceae NK4A136* group” and *Helicobacter* in the M group, and *Helicobacter* in the MP group were found to be predominant. But, in the UT group, *Weissella* and *Staphylococcus* were found to be predominant. *Eubacterium siraeum group*, *Clostridia vadinBB60 group, Erysipelatoclostridium* and *Anaeroplasma* genera were identified to have a significant (FDR p < 0.05) differential abundance in V compared to C and UT groups. While at the species level, the abundance of *Helicobacter mastomyrinus, Massiliomicrobiota timonensis* and *uncultured Anaeroplasma* were identified as significantly low in UT, C, and M compared to V group. Functional categories related to amino acid, carbohydrate, and energy metabolism, cell motility and cell cycle control were dominated overall across all the samples. Methane metabolism was identified as an enriched pathway. For the C group, “Colitis (decrease)” was among the significant (p = 1.81E-6) associations based on the host-intrinsic taxon set.

**Conclusion:**

Combination regimen of minocycline plus methyl prednisolone produces a synergistic anti-inflammatory effect which is part related to alternation in the colonic microbiota composition.

**Supplementary Information:**

The online version contains supplementary material available at 10.1186/s12934-023-02242-8.

## Introduction

The incidence and prevalence rate of the two forms of inflammatory bowel disease (IBD); ulcerative colitis (UC) and Crohn`s disease (CD) continues to rise worldwide and recently in newly industrialized countries [1]. IBD is a multifactorial disease with the involvement of genetic, environmental, immunological, and microbial factors in its pathogenesis. T-helper-1 mediated immune response in the main manifestation of CD [2], while T-helper-2 is the driving force for UC pathogenesis [3]. Various genetic polymorphisms were shown to be involved in the disease pathogenesis and most of them are linked to the function of the immune response towards intestinal microflora such as multi-drug resistance (MDR), toll-like receptors (TLRs), IL-23, IL-10, IL-17, and interferon (IFN)-γ genes [4]. Furthermore, it has been suggested that consuming a westernized type of diet rich in saturated fat and preservatives is an important factor contributing to the disease pathogenesis. Tobacco smoking was shown to be protective for UC, but considered a risk factor for CD [5]. It was also suggested that strict attention to individual hygiene at a young age limits exposure to some microorganisms, which results in inappropriate immune responses towards microorganisms later in life leading to various inflammatory conditions including IBD [6].

The gut ecosystem contains trillions of microorganisms from over 1000 species with the dominant species being from the *Bacteroidetes* and *Firmicutes* phyla [7]. The gut microbiota play crucial roles in health, as it has function in xenobiotic and drug metabolism, nutrition metabolism and production as well as protecting the host from pathogenic bacteria [8]. In regard to colitis, various spontaneous animal models of colitis such as the HLA-B27 transgenic rats or genetically engineered mice deficient in the cytokines IL-2 or IL-10 do not develop colitis or develop a lesser degree of inflammation if they are kept in a germ-free environment, suggesting the importance of the gut microbiota as a driving force of the inflammatory process in the gut [9, 10]. Moreover, the composition of the microbiota population in IBD patients is disturbed with a significant reduction in the numbers of protective commensals like *Firmicutes, Faecalibacterium, Clostridia, Bacteroides* and increased number of pathogenic microorganisms such as adhesive/invasive *Escherichia coli* (AIEC), *Pseudomonas aeruginosa, Mycobacterium, Helicobacter, and Campylobacter* [11]. Also, the beneficial usage of antibiotics (ciprofloxacin and metronidazole) was proven in IBD patients to treat certain conditions like abscess, pouchitis, toxic megacolon, and perianal CD [12].

We recently showed that there was a synergistic anti-inflammatory effect with minocycline and methyl prednisolone treatment in the murine dextran sulfate sodium (DSS) colitis model (paper in press). This was mediated in part through reduced colonic phosphorylation levels of important pro-inflammatory molecules (e.g., p38 MAPK, ERK1/2, Akt, Src, COX2) and molecules involved in the apoptotic machinery (e.g., BAK, and caspase 3). In this report, we extended our findings to determine the effect of mono- or combination therapy on the composition of bacterial population using fecal samples obtained from control (untreated, UT) mice, or mice subjected to colitis (through DSS administration) plus treatment with vehicle (V), minocycline (M), methyl prednisolone (MP), or combination regimen of minocycline plus methyl prednisolone (C).

## Materials and methods

### Animals

BALB/c mice (6–10 weeks old, mean weight 20 g.) were supplied by the Animal Resource Center of the Health Sciences Center at Kuwait University. All animals were kept under standard conditions including controlled temperature (25 °C), a 12-h light–dark cycle and had free access to food and drinking water ad libitum. All experimentations were approved by the Animal Care Committee at Kuwait University Health Sciences Center and conformed to their rules and regulations as described previously (protocol approval number: P11613PT01) [13].

### Dextran sulfate sodium (DSS) colitis model

Colitis was induced in mice by mixing DSS polymers with the drinking water (3.5% w/v, Cat # 160110, MP Biomedicals) given ad libitum [13, 14]. Control: untreated (UT) mice received tap water. In the treatment groups, mice received DSS plus daily intra-peritoneal (i.p) injections of vehicle (V), minocycline (M) (10 mg/kg, Cat# M9511, Sigma), methyl prednisolone (MP) (5 mg/kg, Cat # P6004, Sigma), or combination (C) regimen of minocycline (10 mg /kg) plus methyl prednisolone (5 mg/kg) for 5 days (n = 3 per group). After that, mice were sacrificed, and the severity of colitis was determined by histological assessment, and fecal samples were collected (in Eppendorf tubes with forceps, snap frozen, and stored in − 80 freezer) [15, 16] to determine the bacterial population using fecal 16s metagenomics sequencing, and the taxonomic and functional profiles were also determined.

### *Histological assessment of colitis severity*

Formalin fixed colons were processed for histological assessment and were (blindly) scored by 2 observers using a standard semi-quantitative histology scoring system as previously described [13].

### Metagenomics sequencing and data-analysis

30 ng qualified DNA template and the 16S rRNA fusion primers were added for Polymerase chain reaction (PCR). All PCR products were purified by Agencourt AMPure XP beads, dissolved in Elution Buffer, and eventually labeled to finish library construction. Library size and concentration are detected by Agilent 2100 Bioanalyzer. Qualified libraries are sequenced on the DNBSeq platform according to their insert size.

Raw data are filtered to generate high quality clean reads as follows: (a) truncate reads whose average phred quality values are lower than 20 over a 25 bp sliding window were truncated. Remove reads whose length are %75 of their original lengths after truncation; (b) remove reads that are contaminated by adapter sequences (default parameter: 15 bases overlapped by reads and adapter with maximal 3 bases mismatch allowed); (c) remove reads with ambiguous base (N base); (d) remove low complexity reads (default: reads with 10 consecutive same base). To ensure the removal of barcode sequences from pooling libraries, clean reads were assigned to corresponding samples through alignments (0 base mismatch) against barcode sequences. iTools Fqtools fqcheck v.0.25 [17], cutadapt v.2.6 [18] and readfq v1.0 (https://github.com/cjfields/readfq) were used to perform the filtering.

The raw 16S rRNA gene sequencing data was checked for quality using FastQC v0.10.1 (https://www.bioinformatics.babraham.ac.uk/projects/fastqc). DADA2 pipeline implemented in QIIME2 [19] was used for detecting and correcting Illumina amplicon sequence data. This quality control process filters any ‘phiX’ reads (commonly present in marker gene Illumina sequence data) that are identified in the sequencing data including chimeric sequences, resulting in filtered non-chimeric sequences. No truncation was done for the reads as the sequence quality was good. The quality filtered reads were combined and clustered to identify OTUs or sequence variants (ASVs) with 100% similarity.

The pre-trained classifier (silva-132-99-515-806-nb-classifier.qza) based on 16S rRNA gene sequences was downloaded from SILVA [20] database and used for the classification of the bacterial sequences. These sequences were then used to train the naive-bayes classifier. The q2-feature-classifier plugin implemented in QIIME2 was used to classify the representative sequences using sklearn tool. The classified taxonomies were used for further downstream analysis. The representative sequences (OTUs) were aligned using the q2-phylogeny plugin in qiime2. The mafft algorithm [21] was used to perform the multiple sequence alignment of the representative sequences. The aligned sequences were filtered for the highly variable regions, followed by construction of phylogenetic tree using the FastTree program [22].

The OTU table and corresponding taxa were further used to correlate and analyze across samples and groups using MicrobiomeAnalyst [23]. Briefly, low quality or uninformative features were removed to improve downstream statistical analysis, as described below:

Filtering out the low count features—a minimum count of 4 features were retained, as the features with a very small count in very few samples are likely due to sequencing errors or low-level contaminations (a 20% prevalence filter means at least 20% of its values should contain at least 4 counts).

Low variance filter—features that are close to constant throughout the experiment conditions are unlikely to be associated with the conditions under study. Their variances can be measured using inter-quantile range (IQR), standard deviation or coefficient of variation (CV). The lowest percentage based on the cutoff (10%) were excluded. Normalization was performed before summarizing and comparing the taxonomic profiles and their distribution across the samples and groups. Briefly, the normalization aims to address the variability in sampling depth and the sparsity of the data to enable more biologically meaningful comparisons. During normalization, the data was neither rarified nor transformed, but was scaled using Total Sum Scaling (TSS) factor.

Using the filtered and normalized data further downstream analysis was performed. Abundance plots were generated at various levels of taxa. Rarefaction curve plots were generated to check whether enough sequencing depth was attained for all the samples. Alpha diversity plots were plotted, and the statistical measures were obtained to indicate the richness/evenness of microbiome across samples. Beta-diversity profiling that helps in assessing the microbial diversity differences across samples and groups was performed using Principal Coordinate Analysis (PCoA) analysis, by PERMANOVA statistical measure. Brau-Curtis's index measure was used as distance method for PCoA analysis. Additionally, core microbiome analysis was performed at different levels of taxa that identifies features that remain unchanged in their composition across sample groups. A minimum of 20% prevalence and 0.01% relative abundance was considered for this analysis.

Several correlation analyses were performed to identify any inherited associations or patterns across samples and experimental groups based on the taxa abundance profiles. Clustering was performed using Euclidean distance measure and Ward linkage method, and the heatmaps representing the abundance pattern were generated. Separately, the dendrograms were plotted based on the phylogenetic analysis on samples using either various phylogenetic or non-phylogenetic distance measures. The phylogenetic tree generated using qiime2 was used for dendrogram analysis. Pattern search analysis that helps in identifying or search for a pattern based on correlation analysis was performed using ‘Pearson r’ distance measure available within the MicrobiomeAnalyst [23].

Comparisons were performed using several statistical approaches to identify taxa that have significant differential abundances across experimental groups. Single-factor analysis was conducted using both T-test/ANOVA and edgeR statistical methods. In both methods, the taxa obtained with an adjusted p < 0.05 were considered significant.

Biomarker analysis was performed using Random Forests Classification (RFS) within MicrobiomeAnalyst [23]. Briefly, ensemble learning method used for classification, regression, and other analysis by constructing a multitude of decision trees at training time and outputting the class that is the mode of the classes (classification) of the individual trees. Random forests correct for decision trees habit of over fitting to their training set. The random forests provide the estimation of important variables in the classification of data. Mean Decrease Accuracy is computes proximities between pairs of cases that can be used in clustering, locating outliers, or give interesting views of the data.

### Functional analysis

The functional predictions based on 16S rRNA marker gene data was performed using Tax4Fun [24] integrated within the MicrobiomeAnalyst [23]. Tax4Fun is designed for functional prediction based on minimum 16S-rRNA sequence similarity. Briefly, Tax4Fun uses SILVA annotations and outputs KEGG abundance profiles that include KEGG pathways, modules or EC categories or COG annotations. The functional features appearing in only one sample (considered artifacts) are excluded for further analysis. Feature filtering (prevalence and low variance) similar to that of the taxonomic profiles was considered, and the cumulative sum scaling was used for normalizing the features.

The functional categories were tested for their associations with the experimental factors/groups using globaltest [25] implemented within the MicrobiomeAnalyst [23]. Functional associations enriched with an FDR p < 0.05 were considered significant.

Cluster analysis was performed using several statistical approaches including unsupervised clustering, Principal Compoent Analysis (PCA), and dendrogram, to obtain any inherited functional correlation among the samples within and across groups. Clustering was performed using Euclidean distance measure and Ward linkage method, and the heatmaps representing the abundance pattern were generated. The potential biomarker profiles significantly associated with the metagenomes were obtained using Linear Discriminant Analysis Effect Size (LEfSe) within MicrobiomeAnalyst [23]. It employs the Kruskal–Wallis rank sum test to detect the significant differential abundance with regard to class labels, followed by Linear Discriminant Analysis to evaluate the relevance or effect size of differential abundant features. Features with log LDA scores of more than 1 and FDR adjusted p-value < 0.05 are reported significant.

Comparisons were performed using several statistical approaches to identify significant differential functional associations across experimental groups. Single-factor analysis was conducted using both T-test/ANOVA statistical methods. In both the methods, the associations obtained with an adjusted p < 0.05 were considered significant. Clustering was performed for the significant associations using the functional abundance profiles and the heatmaps were generated using ClustVis. For clustering, rows (KO-IDs) are centered, and unit variance scaling is applied. Both rows and columns (samples) are clustered using Euclidean distance and Ward linkage.

The taxa enrichment analysis (TSEA) was performed to identify biologically or ecologically meaningful patterns by comparing them with a pre-defined taxon set that shares common traits. The “host-intrinsic taxon set” used for comparison included 454 taxon sets associated with host-intrinsic factors such as diseases. The taxa obtained across all samples at the genus/species level were considered for the taxa enrichment analysis. The Over Representation Analysis (ORA) is performed on the list of taxa using the hypergeometric test to evaluate whether a specific Taxon set is represented more than expected by chance. One-tailed p-values are provided after adjusting for multiple testing.

### Statistical analysis

For the histological assessment of colitis severity, data were analyzed using GraphPad Instat and Prism softwares (California, USA). Differences between groups were assessed using one-way ANOVA followed by Bonferroni post-hoc test, with p < 0.05 being regarded as significant.

## Results

### *Effect of minocycline, methyl prednisolone, and combination regimen on colitis severity*

**Fig. 1:**
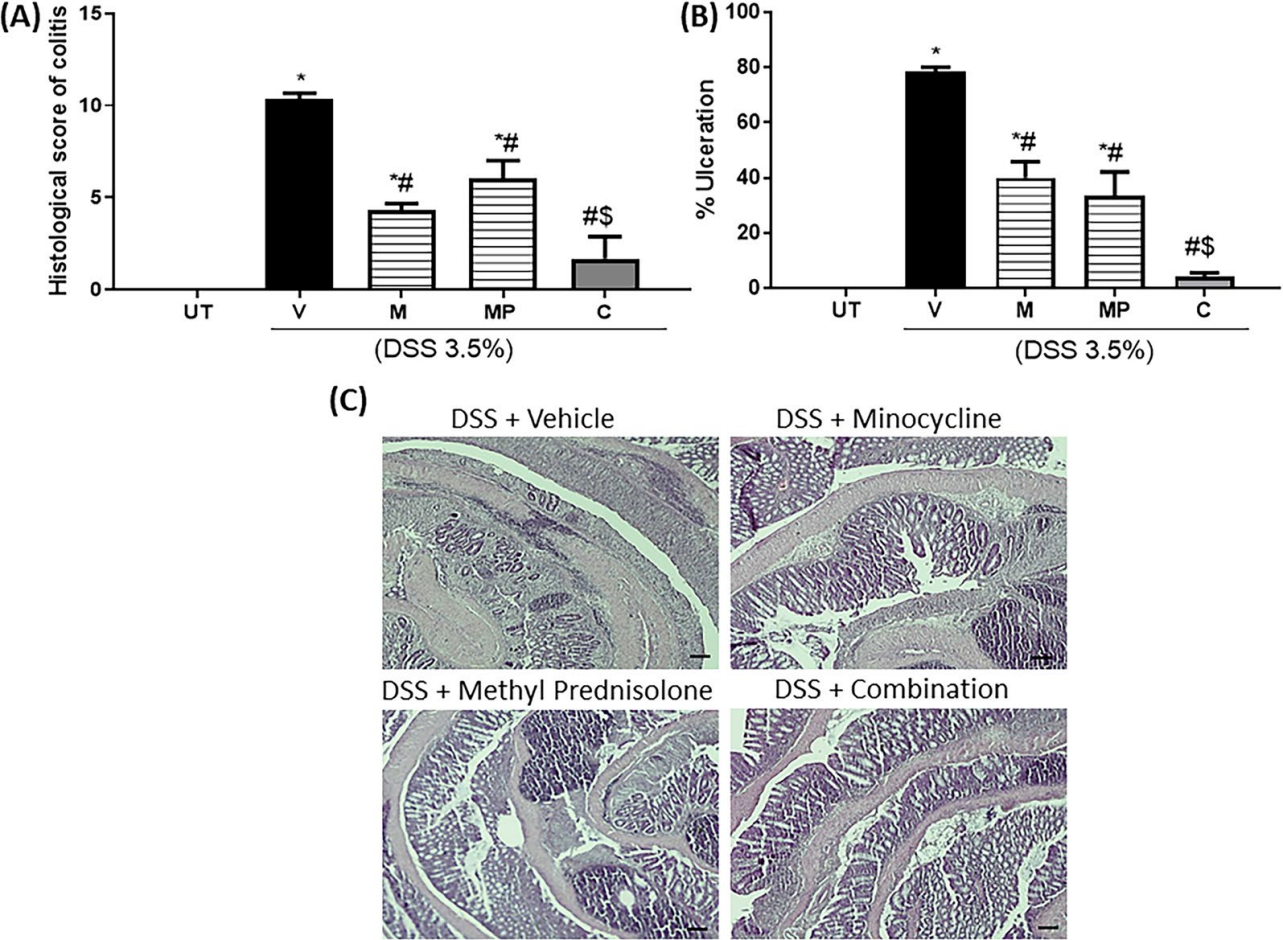
Effect of minocycline, methyl prednisolone, or combination regimen on colitis severity at the histological level. Panel A shows the histological assessment of colitis severity, and panel B shows the % of ulceration in the whole colon section in mice receiving DSS and either vehicle (solid bars), monotherapy (hatched bars), combination regimen (gray bar), and in the UT healthy mice (open bars). Histobars represent means ± SEM for 3 mice in each group. Asterisks denote significant difference from UT mice with p < 0.05 (*), # denotes significant difference from DSS/vehicle-treated mice, $ denotes significant difference from monotherapy with p < 0.05. Panel C is an illustration of colon sections taken from various treatment groups (10 × magnification, bars represent 150μm)

There was a significant increase in the histological score of colitis in DSS/vehicle (V) treated compared to UT group, which was significantly reduced (40–50%) by treatment with minocycline (M) or methyl prednisolone (MP) alone. Combination (C) regimen resulted in a more robust reduction in the histological colitis score (Fig. 1A). The same pattern was also observed regarding the percentage of ulceration involved in the whole colon in the treatment groups (Fig. 1B). Figure 1C shows examples of colon sections taken from the various treatment groups. DSS administration in mice received vehicle treatment resulted in significant destruction of mucosal architecture, transmural immune cell infiltration, submucosal edema, and muscle wall thickness. Treatment with DSS plus minocycline or methyl prednisolone alone significantly reduced the degree of mucosal architecture destruction and the degree of immune cell infiltration and submucosal edema. Treatment with combination regimen eliminated all of these signs of inflammation.

### Metagenomics sequencing and data-analysis

A total of 837 Mb downloadable 16S metagenomics data was obtained after sequencing. After trimming for low-quality reads, on average, 93% of the sequencing data per sample was used for further analysis. A total of 1298 OTUs were obtained, of which 1072 had taxa assigned with a minimum confidence of 85%.

**Fig. 2:**
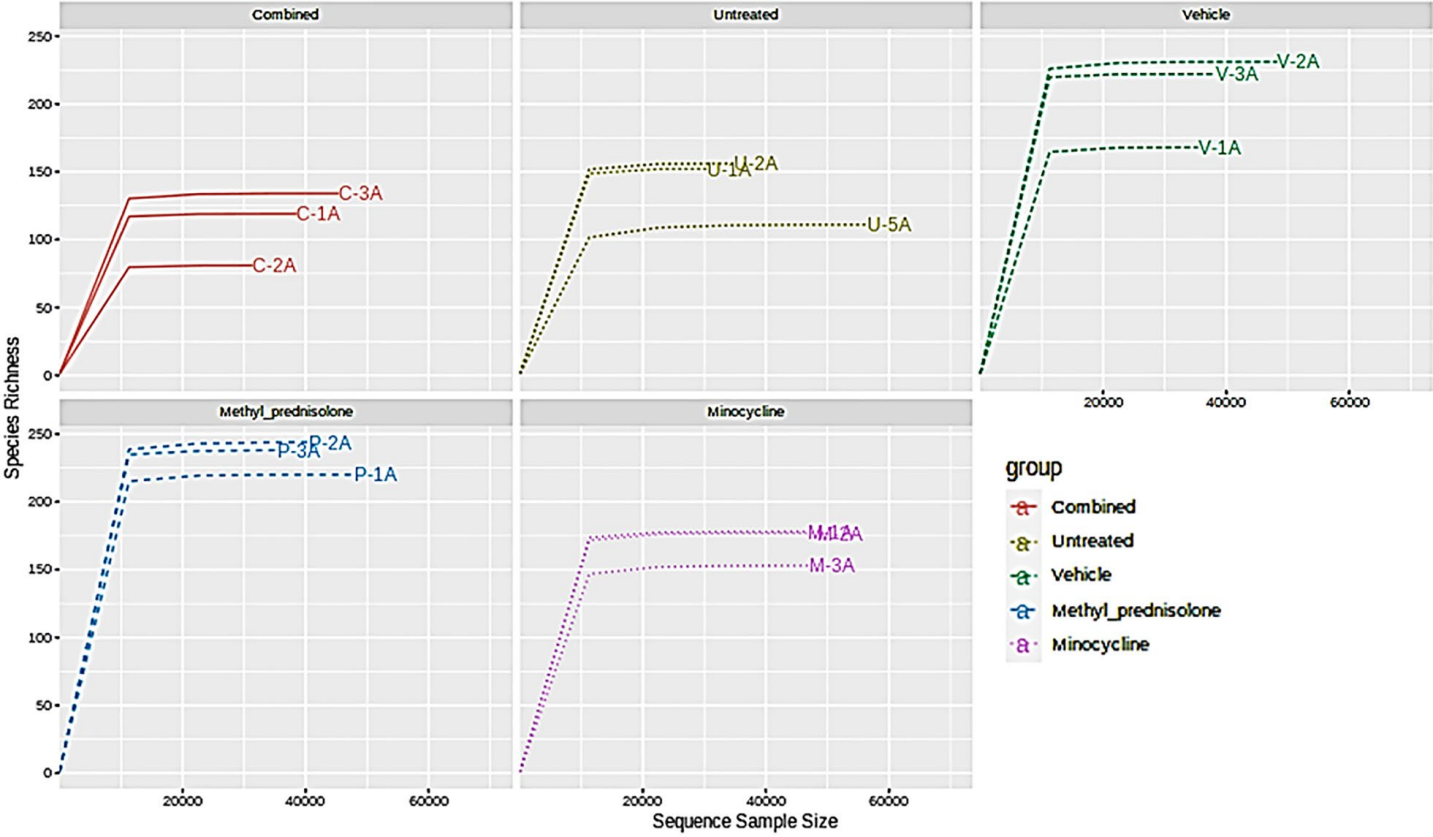
Group-wise rarefaction plots

**Fig. 3:**
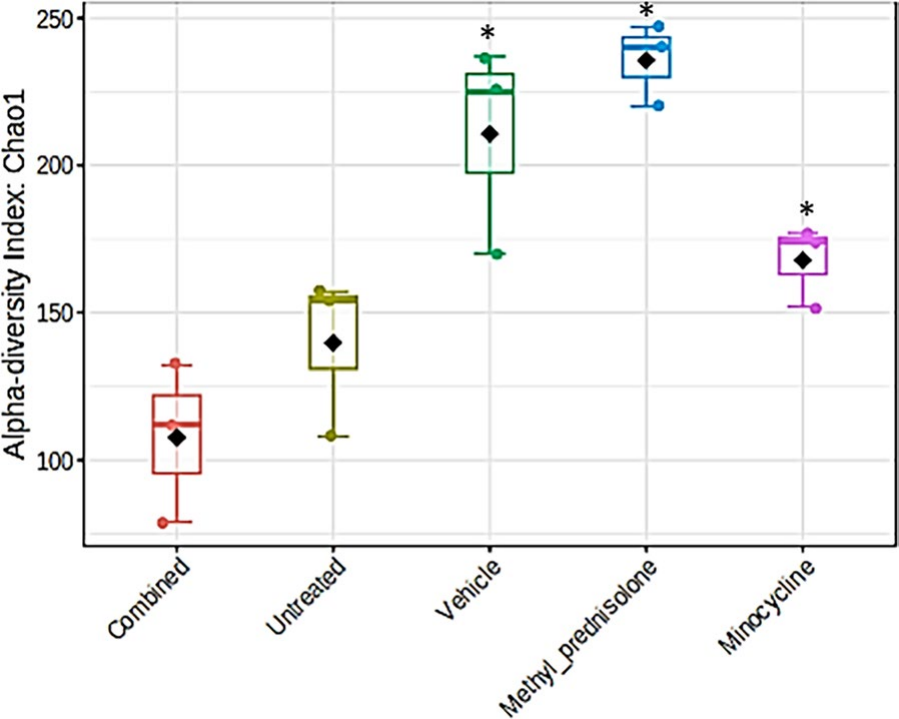
Alpha diversity plot representing Chao1 index. Statistics at the feature level: p-value: 0.00058824; [ANOVA] F-value: 12.882. Asterisks denote significant difference from untreated (UT) mice

All the samples were shown to have enough sequencing depth, Fig. 2. The samples from the MP group represented more diversity or richness of microbial flora compared to other groups. However, two samples from the vehicle-treated group (V2 & V3) also displayed similar richness to MP treatment (Fig. 2). The alpha diversity plots are represented in Fig. 3. The C and the UT groups however displayed less diversity of microflora compared to other groups.

**Fig. 4:**
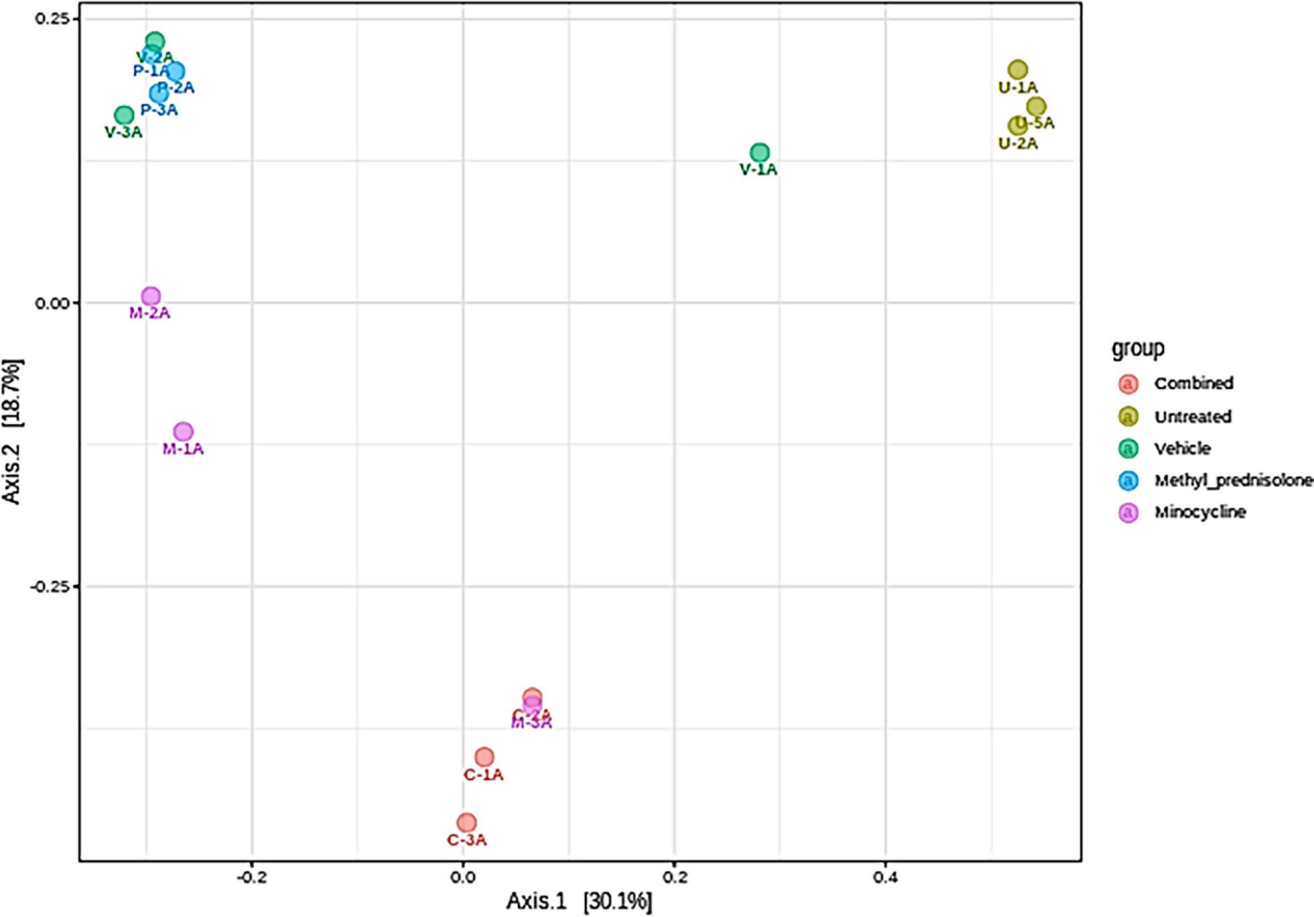
Beta diversity across groups. Statistics at the feature level: PERMANOVA F-value: 2.9043; R-squared: 0.5374; p-value: 0.001

After filtering for low-quality or uninformative features a total of 447 features were retained for the downstream statistical analysis. Based on the beta-diversity plots, a significant correlation of groups was observed at the feature (p-value: 0.00058824) and genus (p-value: 0.00065446) level data. The principal coordinate analysis (PCoA) or the beta diversity plots indicating diversity across groups are represented in Fig. 4.

**Fig. 5:**
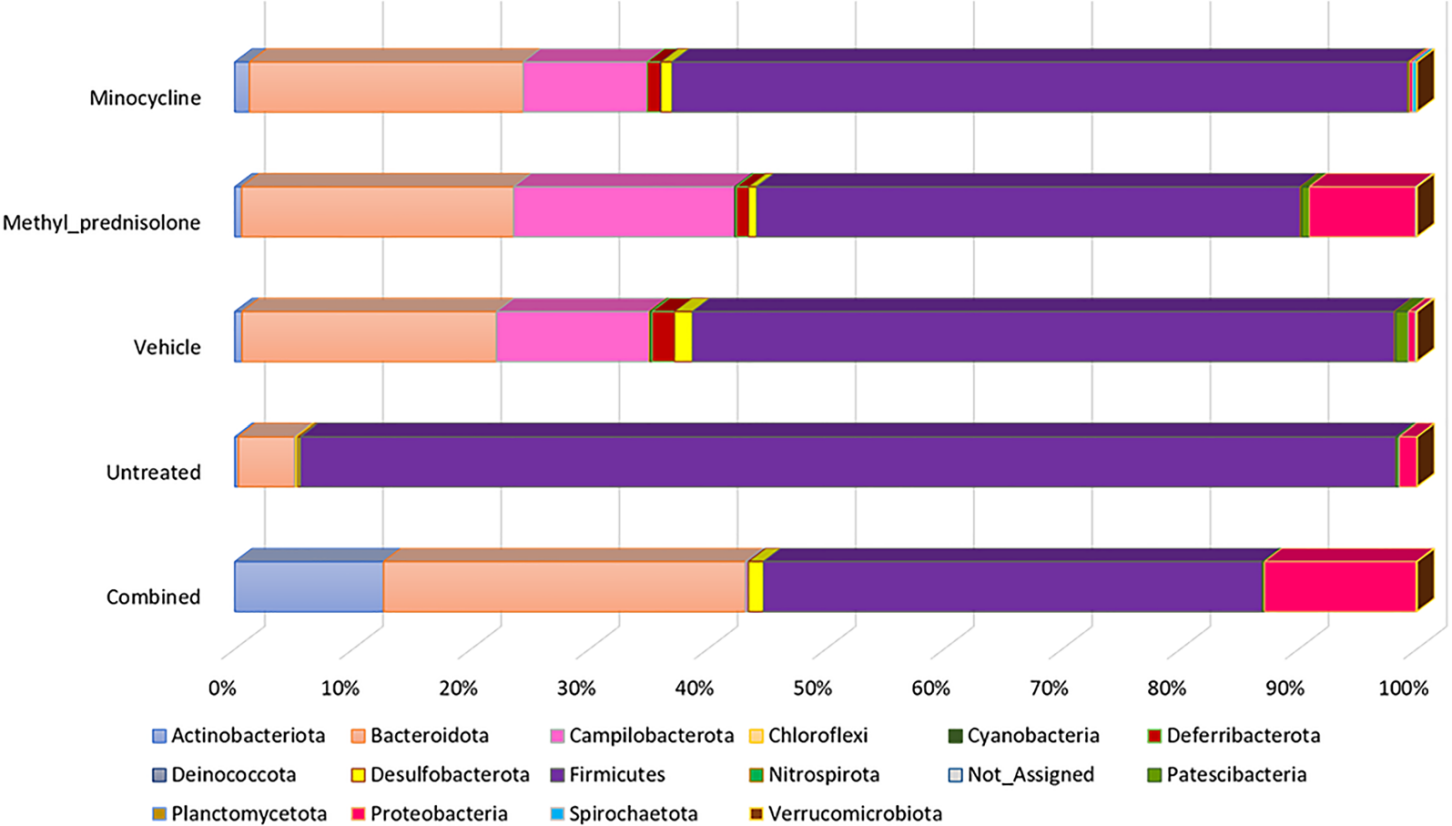
Abundance profiles at phylum level

Overall, *Firmicutes* were found to be predominant in all the samples, followed by bacteria from the phylum *Bacteroidota*. However, the abundance of *Firmicutes* was found to be altered in all the treatment groups compared to UT group. A relatively higher percentage of *Actinobacteriota* was observed in the samples from the C group (Fig. 5). Whereas *Proteobcateria* was found to be higher in the case of C and MP group. Similarly, the abundance of *Deferribacterota* and *Campilobacterota* were comparatively lower in UT and C group of samples.

**Fig. 6:**
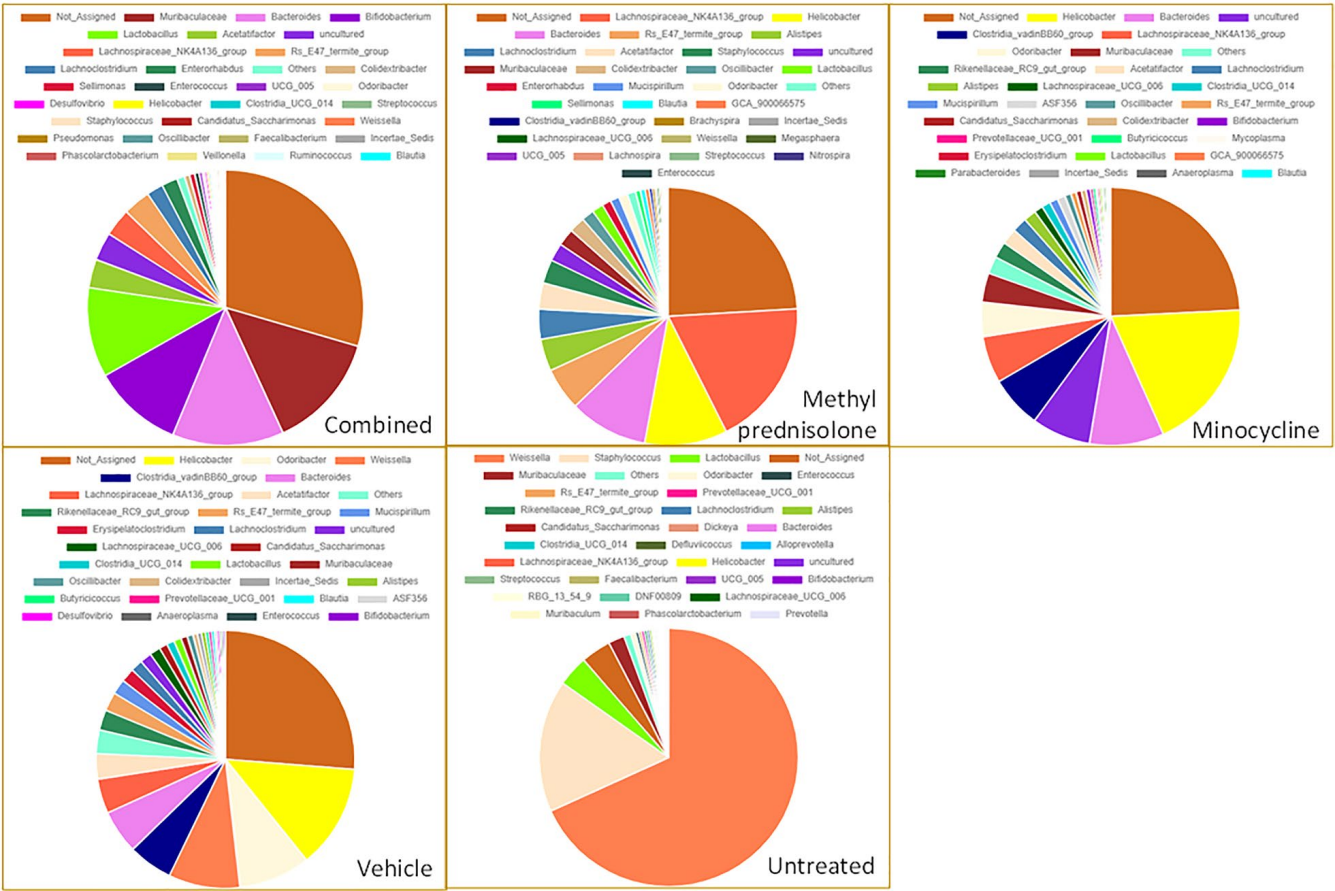
Abundance profiles at genus level

At the genus level, *Muribaculaceae*, *Bacteroides*, *Bifidobacterium*, and *Lactobacillus* were found to be predominant (> 10%) in the samples treated with both the drugs (C group, Fig. 6). Whereas the genus “*Lachnospiraceae NK4A136 group*” and *Helicobacter* in the M group, and *Helicobacter* in the MP group were found to be predominant with > 10% abundance. Interestingly, bacteria from the genus *Helicobacter* were found to have > 10% abundance even in the samples with V group. But, in the UT group, bacteria from the genus *Weissella* (68%) and *Staphylococcus* (16%) were found to be predominant.

**Fig. 7:**
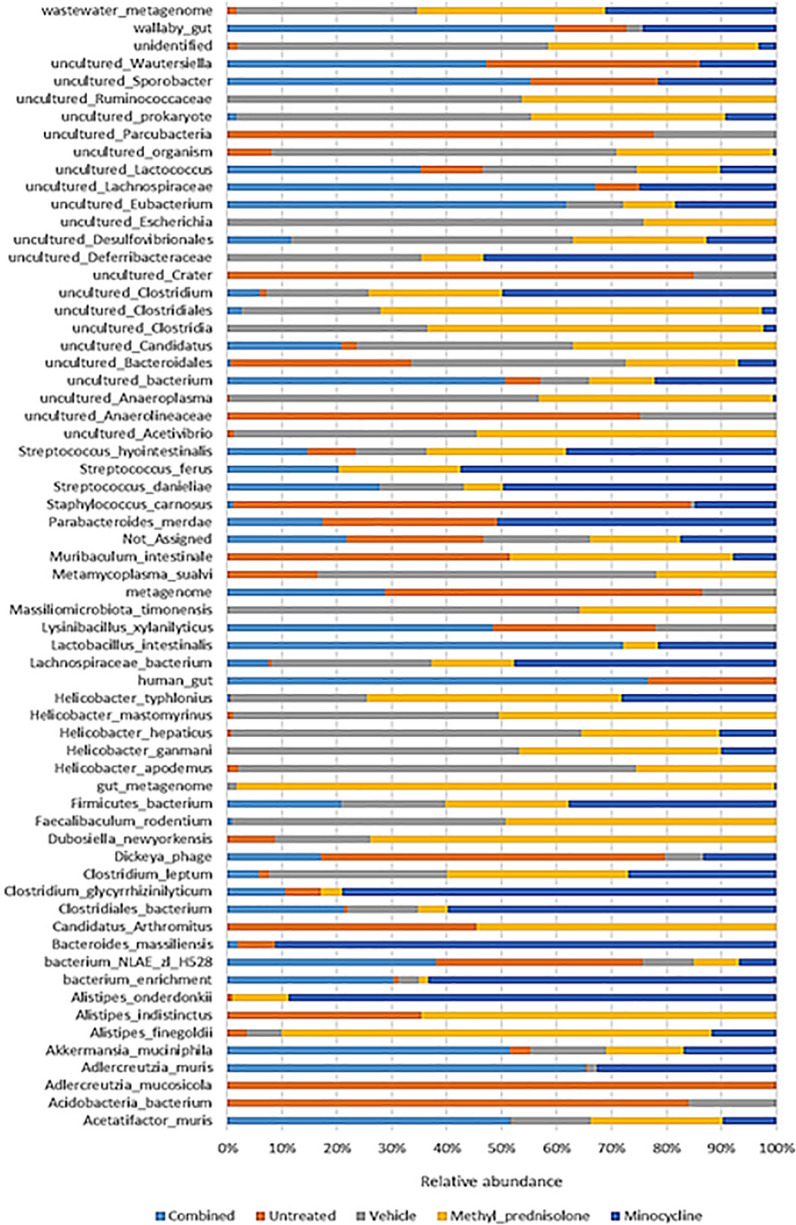
Abundance profiles at species level

Bacteria from the species *Clostridiales bacterium* and *Helicobacter typhlonius* were identified with more than 10% abundance in the M samples, whereas only *Helicobacter typhlonius* species was identified in the case of samples from MP group (Fig. 7). But, in the C group, the bacteria with > 10% abundance were either not assigned to any species or from uncultured bacteria. However, *Acetatifactor muris* and* Adlercreutzia muris* were comparatively more abundant in C group. In the UT group, *Staphylococcus carnosus* (16%) were found to be predominant. These bacteria were also found to be higher abundant in M compared to MP and V group samples. Interestingly, in the samples from V group, *Clostridiales bacterium*, *Helicobacter apodemus* and *Helicobacter ganmani* were found to be higher compared to all other groups. Noticeably, several uncultured species were specifically found in each of the groups. For example, *uncultured Wautersiella* and *uncultured Sporobacter* were identified in UT, and C and M groups. Whereas *uncultured Ruminococcaceae* was found in V and MP groups. Likewise, a few other species were shared across only some groups. For example, *Candidatus Arthromitus* was found in higher abundance comparatively in UT and MP, and *Faecalibaculum rodentium* and *Massiliomicrobiota timonensis* in V and MP groups. The species from *Alistipes* showed distinct distribution across groups. While *Alistipes finegoldii* was found to be comparatively high post MP treatment, *Alistipes onderdonkii* was dominated in M group, and *Alistipes indistinctus* showed higher abundance, comparatively in UT and MP groups. The species from wastewater metagenome were present in all the groups, but not in the C group.

**Fig. 8:**
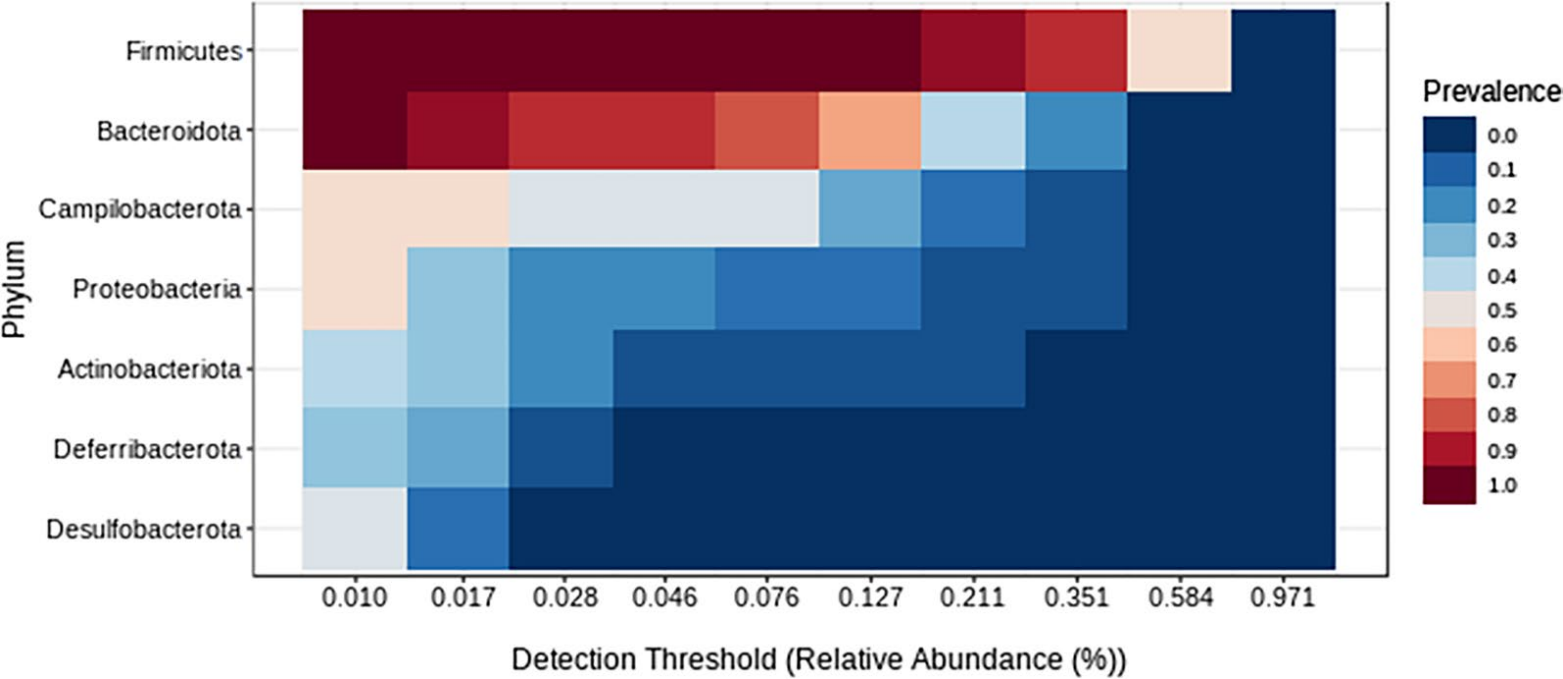
Core microbiome at the phylum associated with all the samples. The set of taxa detected at a high fraction of the population with > 20% prevalence and > 0.01% relative abundance

The core microbiome analysis identified the taxa that are unchanged across the samples in each group. At the phylum level, *Firmicutes* and *Bacteroidota* were identified as core taxa in all the samples (Fig. 8). While both the phyla remained to be as core taxa in each of the individual groups, additionally, *Actinobacteriota* was identified for the samples in C group, *Campilobacterota* and *Deferribacterota* were identified for M group, *Campilobacterota* and *Proteobacteria* for the MP group with at least 50% prevalence. And, bacteria from the phyla *Campilobacterota*, *Deferribacterota* and *Desulfobacterota* were identified as core taxa additionally in the V group. Whereas *Proteobacteria* was found to be core taxa along with the *Firmicutes* and *Bacteroidota* in the UT group of samples (Supplementary Figures).

**Fig. 9:**
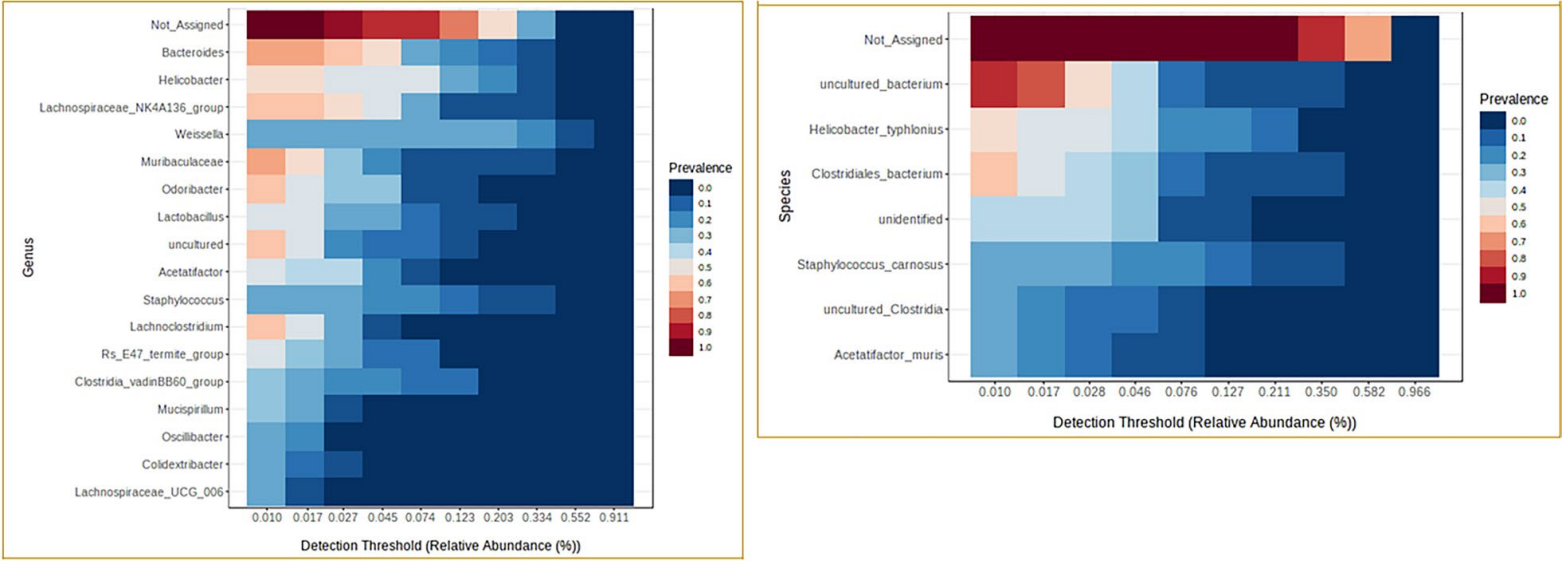
Core microbiome associated with all the samples at the genus and species level. The set of taxa detected at a high fraction of the population with > 20% prevalence and > 0.01% relative abundance

**Fig. 10:**
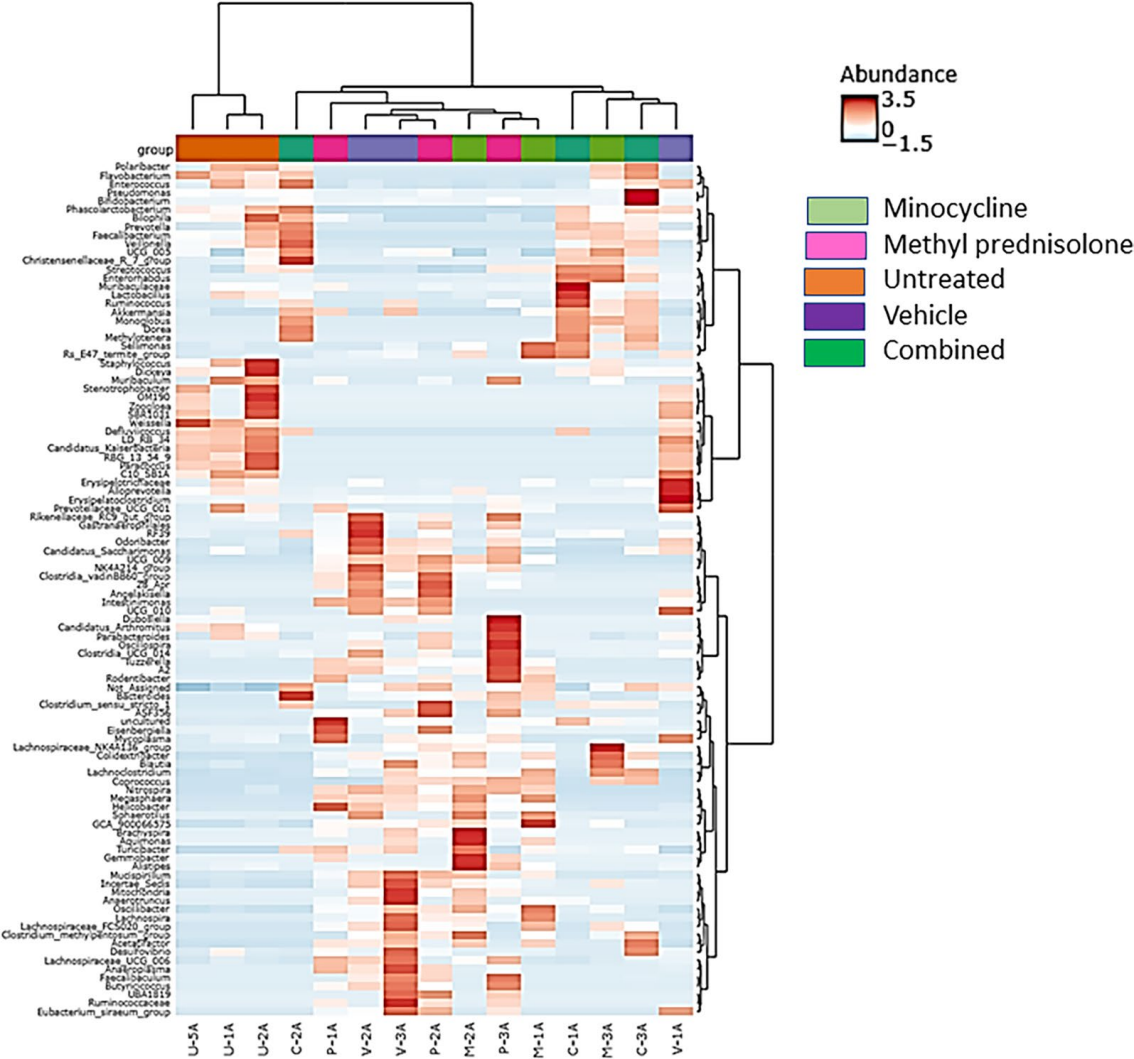
Heatmap representing genus level distribution correlation across samples. Euclidean distance measure and Ward linkage methods were used for clustering

At the genus level, *Bacteroids*, *Helicobacter*, *Lachnospiraceae NK4A136 group*, *Muribaculaceae*, *Odoribacter*, and *Lachnoclostridium* were identified as core taxa in samples across all the groups with at least 50% prevalence (Fig. 9). In addition to these core genus, *Lactobacillus*, *Bifodobacterium* and *Enterorhabdus* were identified in C group, Rs *E47 termite group*, *Acetatifactor*, *Alistipes*, *Colidextribacter*, *Oscillibacter*, *Mucispirillum* were identified for the M group, and, *Clostridia vadin BB60 group*, *Rikenellaceae RC9 gut group*, *Acetatifactor*, *Lachnospiraceae UCG 006*, *ASF356* were identified for the MP samples. Whereas, in the vehicle treated samples (V), additionally, *Clostridia vadin BB60 group*, *Acetatifator*, *Rikenellaceae RC9 gut group*, *Mucispirillum*, and *Lachnospiraceae UCG 006* were identified as core taxa, and, *Weissella*, *Staphylococcus* and *Lactobacillus* for the untreated samples (Supplementary Figures). At the genus level, the taxa from UT samples were grouped into a separate cluster (Fig. 10).

**Fig. 11:**
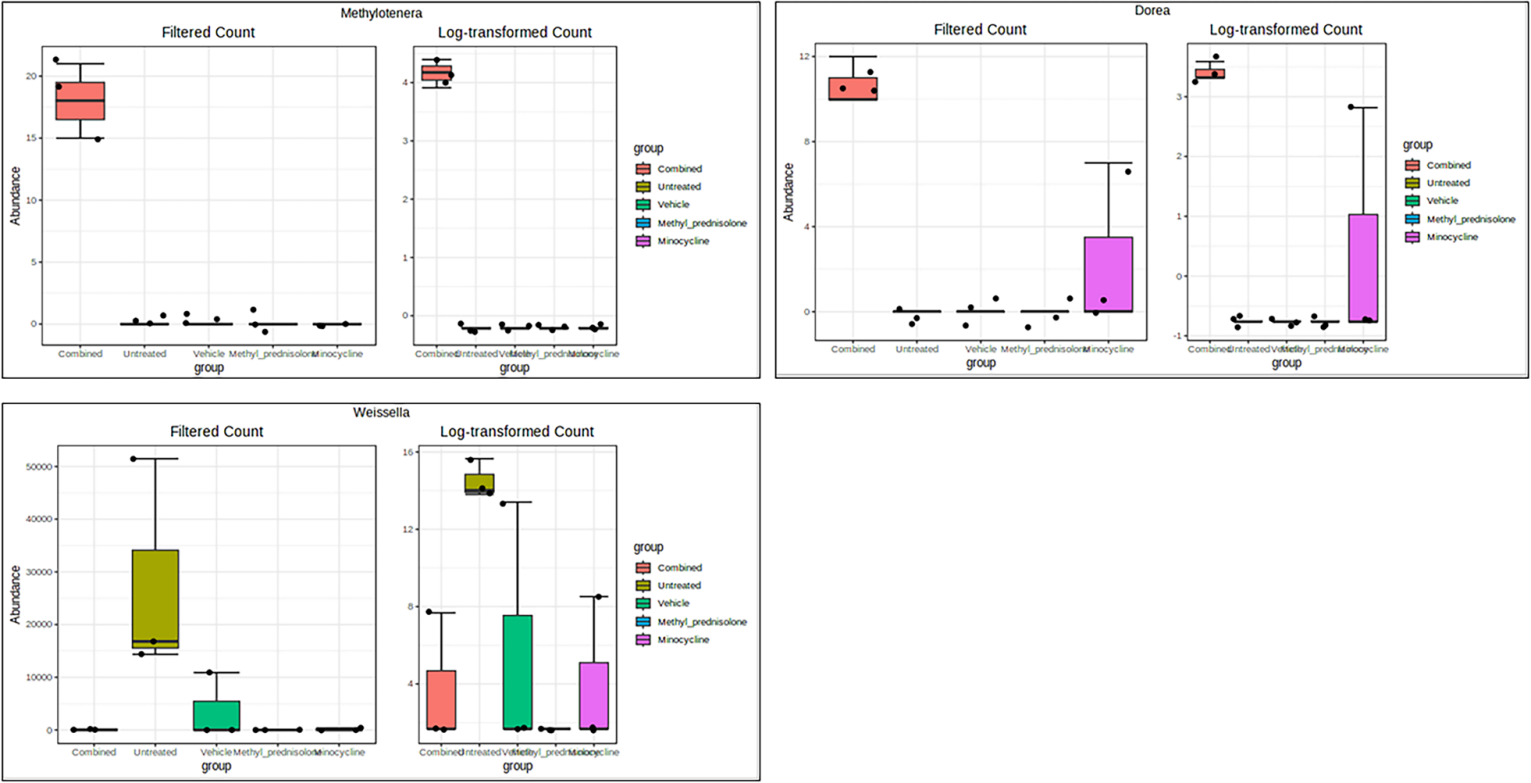
Box plots of significant differential taxa at genus level. Taxa with significantly (FDR p < 0.05) differential abundance levels across groups are represented

The univariate analysis using T-test/ANOVA identified three genus, viz., *Methylotenera*, *Dorea* and *Weissella* to have a significant differential abundance (adjusted p < 0.05) in one group compared to any other group (Fig. 11). While the abundance of bacteria from *Methylotenera* and *Dorea* was of significantly high in the C group, bacteria from the genus *Weissella* were high the in UT group. Using multiple regression analysis, several taxa were identified to be significantly different in their abundance at both genus and species level in treatment groups compared to vehicle/UT groups, Table 1.

**Table 1:** Differential abundant genera identified across comparisons

	TAXA NAME	LOG2FC	LOGCPM	PVALUES	FDR
C vs. U	*Dorea*	1.97	0.237	8.62E-06	0.00348
	*DNF00809*	− 1.86	0.32	0.000171	0.00866
	*Rikenellaceae_RC9_gut_group*	− 7.12	1.16	0.000107	0.00866
	*RBG_13_54_9*	− 1.34	0.27	0.000558	0.015
	*Monoglobus*	1.4	0.291	0.000727	0.0173
	*Candidatus_Kaiserbacteria*	− 1.3	0.312	0.0019	0.0285
	*Paracoccus*	− 1.47	0.369	0.00263	0.0295
	*Not_Assigned*	2.6	0.683	0.00344	0.0347
C vs. V	*Dorea*	1.97	0.237	8.62E-06	0.00138
	*Eubacterium_siraeum_group*	− 5.32	0.677	1.37E-05	0.00138
	*Rikenellaceae_RC9_gut_group*	− 9.16	1.16	1.27E-05	0.00138
	*Erysipelatoclostridium*	− 6.04	1.17	0.000413	0.0185
	*Clostridia_vadinBB60_group*	− 7.43	1.54	0.000712	0.0226
	*Monoglobus*	1.4	0.291	0.000727	0.0226
	*Odoribacter*	− 5.68	1.24	0.00104	0.0262
	*Anaeroplasma*	− 5.91	1.34	0.00133	0.0316
P vs. U	*Clostridia_vadinBB60_group*	8.99	1.54	0.000167	0.00866
	*DNF00809*	− 1.86	0.32	0.000171	0.00866
	*ASF356*	6.28	1.26	0.000551	0.015
	*RBG_13_54_9*	− 1.34	0.27	0.000558	0.015
	*Eisenbergiella*	2.76	0.563	0.000619	0.0156
	*Anaeroplasma*	5.86	1.34	0.00141	0.0284
	*Candidatus_Kaiserbacteria*	− 1.3	0.312	0.0019	0.0285
	*GCA_900066575*	3.08	0.74	0.00196	0.0285
	*Staphylococcus*	− 8.11	1.93	0.00183	0.0285
	*uncultured*	6.69	1.61	0.00192	0.0285
	*Weissella*	− 12.9	3.11	0.00202	0.0285
	*Defluviicoccus*	− 1.35	0.331	0.00222	0.0286
	*Paracoccus*	− 1.47	0.369	0.00263	0.0295
	*Rodentibacter*	1.94	0.484	0.00253	0.0295
	*Not_Assigned*	2.6	0.683	0.00343	0.0347
P vs. V	*Eisenbergiella*	2.76	0.563	0.000619	0.0226
	*Eubacterium_siraeum_group*	− 3.19	0.677	0.000821	0.0237
	*Rodentibacter*	1.94	0.484	0.00253	0.0487
M vs. U	*DNF00809*	− 1.86	0.32	0.000171	0.00866
	*GCA_900066575*	3.89	0.74	0.000372	0.015
	*RBG_13_54_9*	− 1.34	0.27	0.000558	0.015
	*Rikenellaceae_RC9_gut_group*	− 5.93	1.16	0.000443	0.015
	*Lachnospiraceae_NK4A136_group*	7.14	1.56	0.00103	0.022
	*Candidatus_Kaiserbacteria*	− 1.3	0.312	0.0019	0.0285
	*Colidextribacter*	5.91	1.45	0.00226	0.0286
	*Defluviicoccus*	− 1.35	0.331	0.00222	0.0286
	*Paracoccus*	− 1.47	0.369	0.00263	0.0295
	*Not_Assigned*	2.68	0.683	0.00283	0.0309
M vs. V	*Eubacterium_siraeum_group*	− 5.32	0.677	1.37E-05	0.00138
	*Rikenellaceae_RC9_gut_group*	− 7.97	1.16	4.20E-05	0.00283
	*Erysipelatoclostridium*	− 6.04	1.17	0.000413	0.0185
	*Candidatus_Saccharimonas*	− 4.54	1.14	0.00252	0.0487
V vs. U	*Eubacterium_siraeum_group*	4.83	0.677	3.14E-05	0.00635
	*DNF00809*	− 1.86	0.32	0.000171	0.00866
	*Clostridia_vadinBB60_group*	7.86	1.54	0.00047	0.015
	*Erysipelatoclostridium*	5.33	1.17	0.00102	0.022
	*Anaeroplasma*	5.58	1.34	0.00197	0.0285
	*Not_Assigned*	2.82	0.683	0.00204	0.0285
	*Incertae_Sedis*	4.41	1.16	0.00342	0.0347
	*RBG_13_54_9*	− 1.01	0.27	0.00386	0.038
	*Lachnospiraceae_NK4A136_group*	5.61	1.56	0.00493	0.0474
	*Staphylococcus*	− 6.88	1.93	0.00518	0.0486

Taxa common to the treatment group (Minocycline or Methyl Prednisolon or combination) vs. vehicle-treated and untreated vs. vehicle-treated group samples are represented in bold

**Fig. 12:**
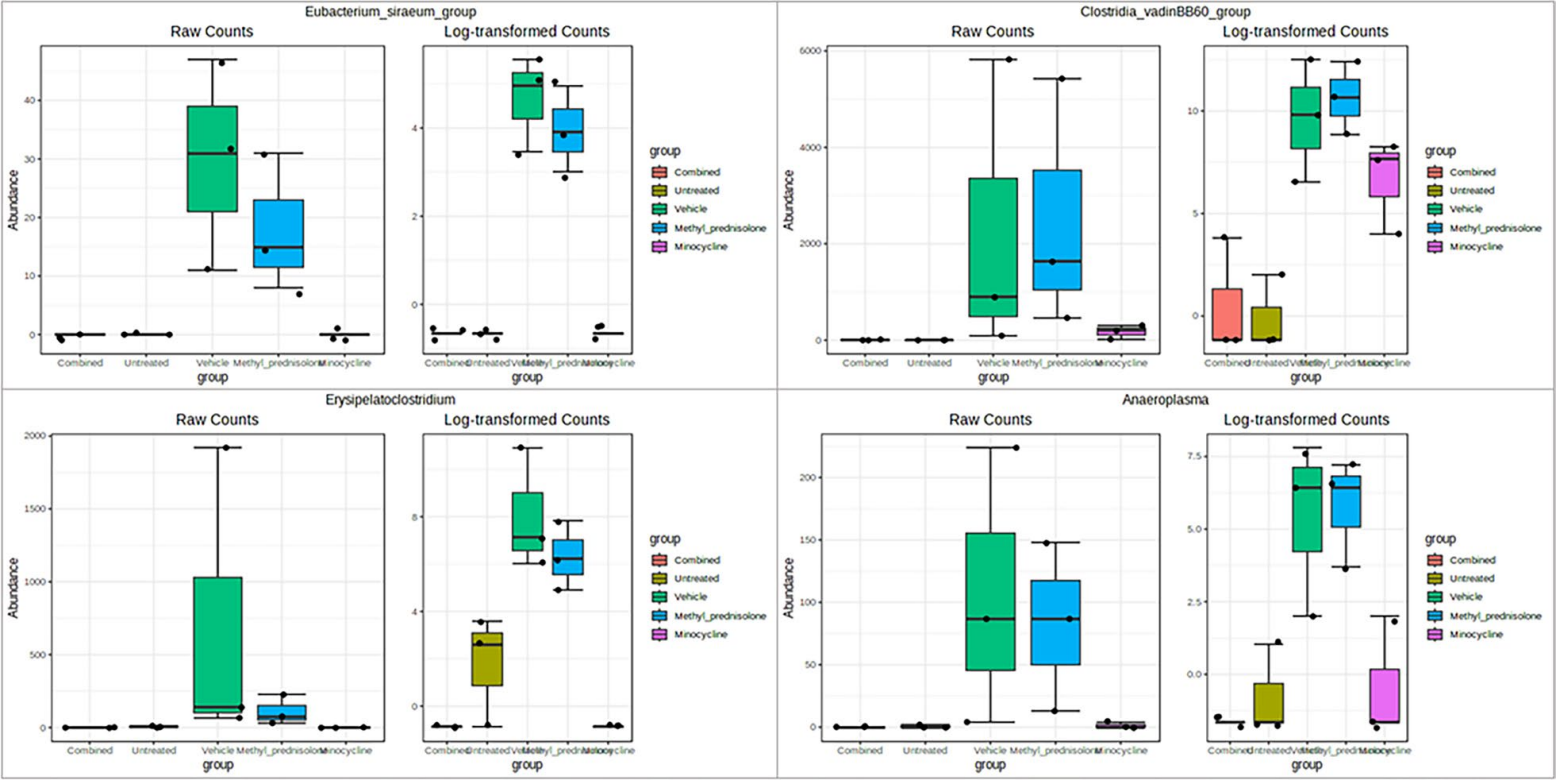
Box plots of significant differential taxa at genus level. Taxa with significantly (FDR p < 0.05) differential abundance levels in Combination compared to vehicle and untreated groups are represented

At the genus level, *Eubacterium siraeum group*, *Clostridia vadinBB60 group, Erysipelatoclostridium* and *Anaeroplasma* were identified to have a significant (FDR p < 0.05) differential abundance in the samples from the C group compared to V group. Similar differences were observed in the UT samples compared to the vehicle-treated group of samples (Fig. 12). These bacteria were in high abundance in vehicle-treated (colitis) compared to the UT and combined-treatment group (C). Interestingly, none of these were identified to have significant differences in their abundance when compared to untreated. Likewise, *Eubacterium siraeum group*, and *Erysipelatoclostridium* genera were identified in Methyl prednisolone-treated samples (M), and *Eubacterium siraeum group* in Minocycline treated samples (M) to be significantly different compared to vehicle-treated (V) samples.

**Fig. 13:**
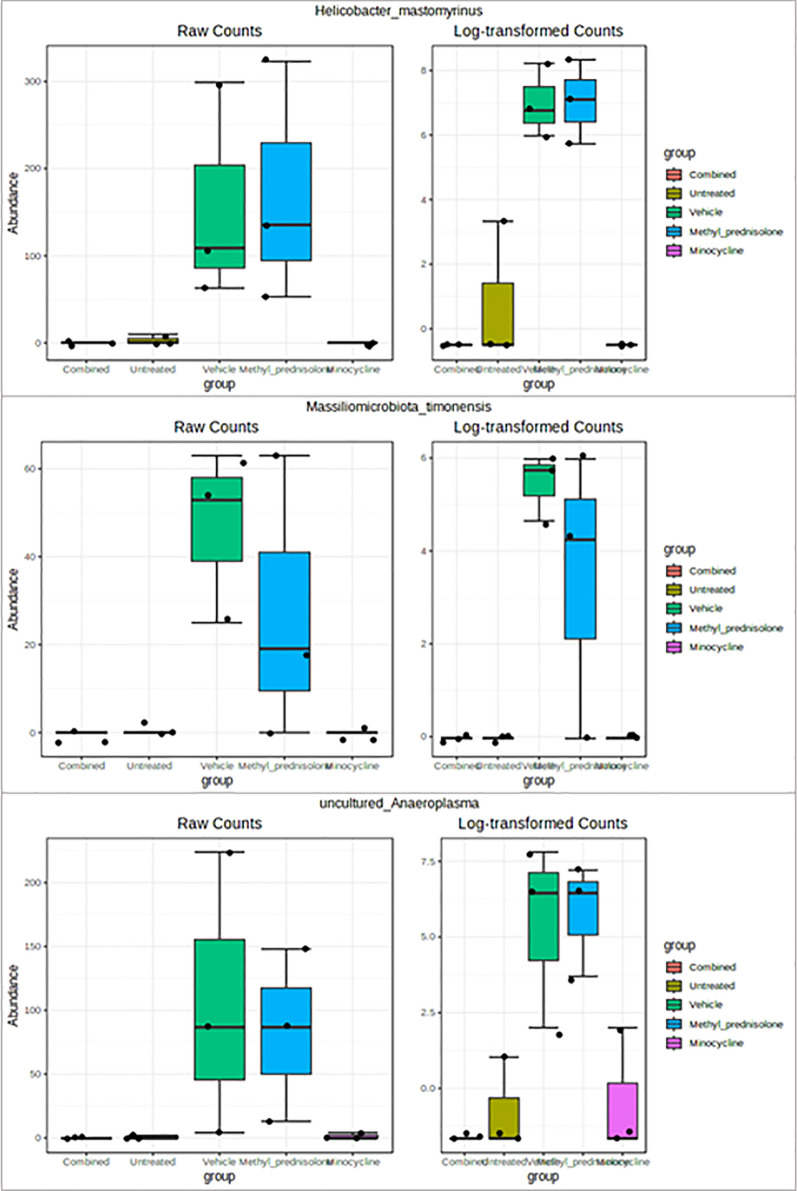
Box plots of significant differential taxa at species level. Taxa with significantly (FDR p < 0.05) differential abundance levels in Combination compared to vehicle and untreated groups are represented

While at the species level, the abundance of *Helicobacter mastomyrinus, Massiliomicrobiota timonensis* and *uncultured Anaeroplasma* was found to be significantly low in UT as well as in the C and M groups of samples compared to vehicle-treated samples, Table 2 and Fig. 13. However, none were identified as significantly different in the MP group compared to V group samples.

**Table 2:** Differential abundant species identified across comparisons

	TAXA NAME	LOG2FC	LOGCPM	PVALUES	FDR
C vs. U	*Adlercreutzia_mucosicola*	− 1.86	0.286	6.79E-05	0.004
	*uncultured_actinobacterium*	− 1.85	0.317	0.000166	0.004
	*uncultured_organism*	− 7.03	1.26	0.000237	0.00522
	*Lactobacillus_intestinalis*	2.77	0.618	0.00116	0.0181
	*uncultured_Parcubacteria*	− 1.31	0.312	0.00184	0.0195
	*Adlercreutzia_muris*	5.53	1.58	0.00573	0.0432
C vs. V	*Helicobacter_mastomyrinus*	− 4.46	0.662	5.14E-05	0.00339
	*uncultured_organism*	− 9.07	1.26	2.94E-05	0.00339
	*unidentified*	− 7.8	1.16	5.13E-05	0.00339
	*Lactobacillus_intestinalis*	2.76	0.613	0.00114	0.0351
	*uncultured_Anaeroplasma*	− 5.91	1.34	0.00133	0.0351
	*Massiliomicrobiota_timonensis*	− 2.23	0.556	0.00245	0.0404
P vs. U	*Adlercreutzia_mucosicola*	− 1.86	0.286	6.79E-05	0.004
	*Helicobacter_mastomyrinus*	4.11	0.658	9.65E-05	0.004
	*uncultured_actinobacterium*	− 1.85	0.317	0.000166	0.004
	*gut_metagenome*	7.82	1.69	0.000941	0.0181
	*uncultured_Clostridiales*	5.1	1.12	0.00106	0.0181
	*uncultured_Clostridium*	2.93	0.64	0.001	0.0181
	*uncultured_Anaeroplasma*	5.87	1.34	0.00139	0.0193
	*Helicobacter_typhlonius*	9.42	2.3	0.00213	0.0195
	*metagenome*	− 1.35	0.33	0.00215	0.0195
	*Staphylococcus_carnosus*	− 8.09	1.97	0.00212	0.0195
	*uncultured_Clostridia*	5.84	1.37	0.00165	0.0195
	*uncultured_Parcubacteria*	− 1.31	0.312	0.00184	0.0195
	*unidentified*	4.64	1.17	0.00264	0.0225
P vs. V	None				
M vs. U	*Adlercreutzia_mucosicola*	− 1.86	0.286	6.79E-05	0.004
	*uncultured_actinobacterium*	− 1.85	0.317	0.000166	0.004
	*uncultured_Clostridium*	3.9	0.64	0.000117	0.004
	*Lachnospiraceae_bacterium*	5.6	1.25	0.00115	0.0181
	*metagenome*	− 1.35	0.33	0.00215	0.0195
	*uncultured_Parcubacteria*	− 1.31	0.312	0.00184	0.0195
	*Firmicutes_bacterium*	3.74	1.02	0.00432	0.0356
M vs. V	*Helicobacter_mastomyrinus*	− 4.46	0.662	5.14E-05	0.00339
	*uncultured_organism*	− 5.96	1.26	0.000804	0.0303
	*Massiliomicrobiota_timonensis*	− 2.23	0.556	0.00245	0.0404
	*unidentified*	− 4.67	1.16	0.0024	0.0404
	*uncultured_Anaeroplasma*	− 5.17	1.34	0.00319	0.0496
V vs. U	*Adlercreutzia_mucosicola*	-1.86	0.286	6.79E-05	0.004
	*Helicobacter_mastomyrinus*	4.12	0.658	9.48E-05	0.004
	*uncultured_actinobacterium*	− 1.85	0.317	0.000166	0.004
	*uncultured_Desulfovibrionales*	3.44	0.777	0.00128	0.0187
	*uncultured_Anaeroplasma*	5.57	1.34	0.00198	0.0195
	*unidentified*	4.96	1.17	0.00171	0.0195
	*Massiliomicrobiota_timonensis*	2.21	0.55	0.00242	0.0213
	*Helicobacter_typhlonius*	8.27	2.3	0.00485	0.0388
	*Staphylococcus_carnosus*	− 6.92	1.97	0.00562	0.0432
	*Lachnospiraceae_bacterium*	4.3	1.25	0.00619	0.0454

Taxa common to the treatment group (Minocycline or Methyl Prednisolon or combination) vs. vehicle-treated and untreated vs. vehicle-treated group samples are represented in bold

### Functional analysis

**Fig. 14:**
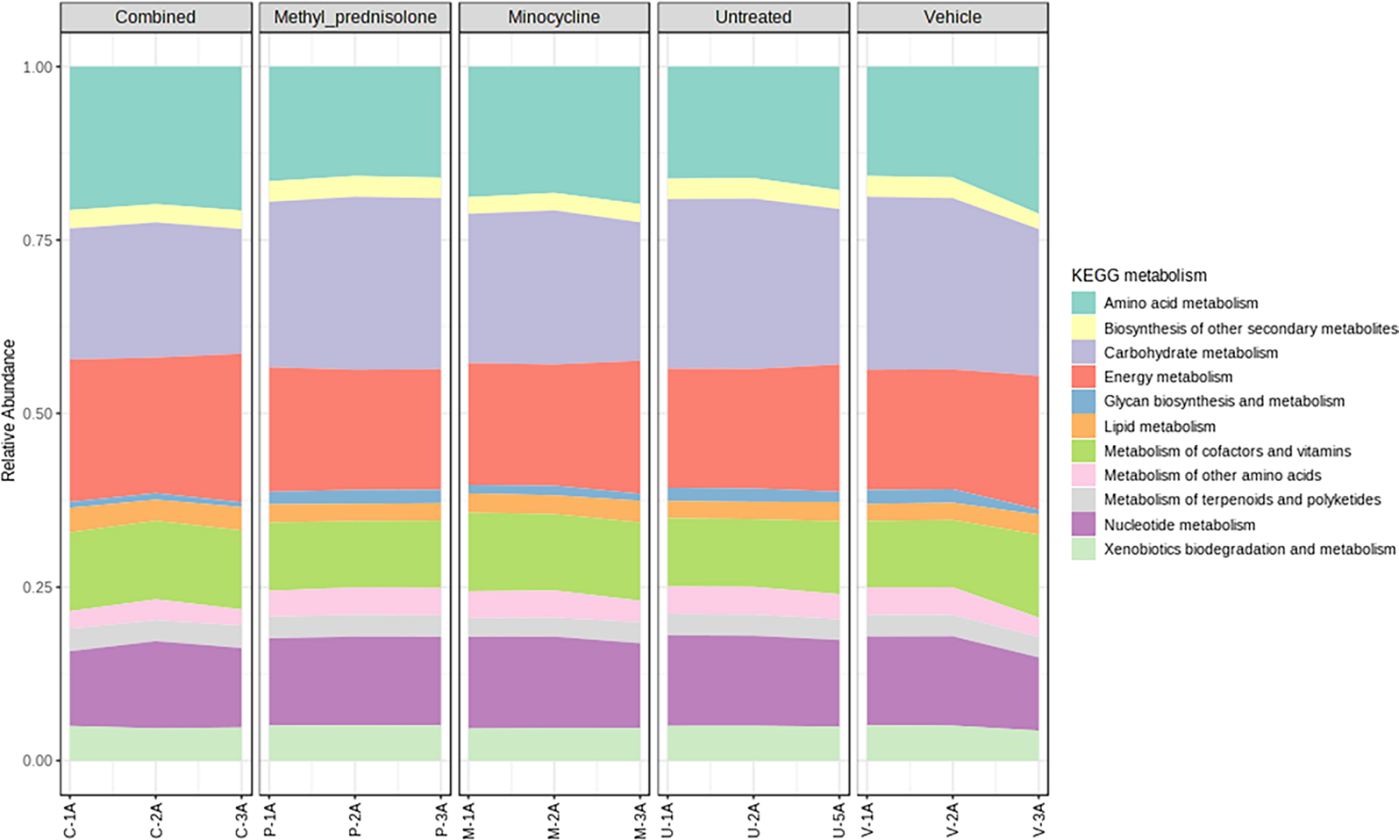
Relative abundance of the functional profiles across groups/samples

A total of 2,839 KEGG Ortholog (KO) IDs were obtained from Tax4Fun analysis. A total of 2,064 functional features remained after filtering. The functional profiling data plots representing KEGG metabolism across groups is presented in Fig. 14. Functional categories related to amino acid, carbohydrate, and energy metabolism were dominated overall across all the samples.

**Fig. 15:**
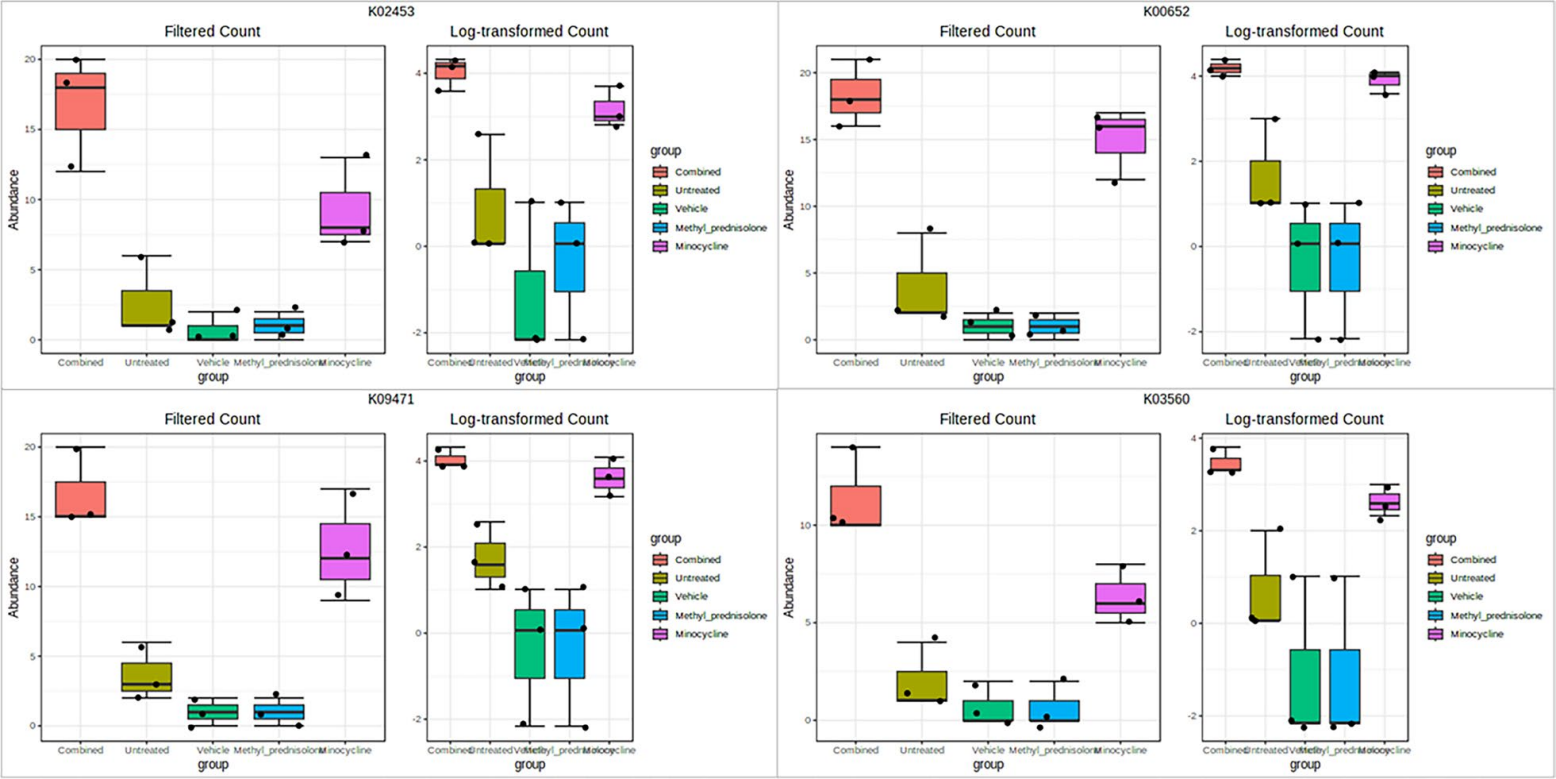
Box plots representing differential functional KO IDs. Plots for the top 4 significant (FDR p < 0.005) KO IDs are represented

Univariate differential analysis with T-test/ANOVA identified 615 KO IDs significantly different in one group compared to all other groups with an FDR p < 0.05. The box plots of the top 4 KO IDs are represented in (Fig. 15). The network mapping of these IDs resulted in the identification of significantly enriched functional profiles (pathways and modules) with FDR p-value < 0.05, Table 3. Methane metabolism was identified as an enriched pathway and “Methanogenesis, CO2 =  > methane”, “F420 biosynthesis, archaea”, “Acetyl-CoA pathway, CO2 =  > acetyl-CoA” and “Methanogenesis, acetate =  > methane” were identified as significantly enriched KEGG modules.

**Table 3:** Enriched KEGG mappings for the significantly different KO abundances

		TOTAL	EXPECTED	HITS	PVAL	FDR
Pathway	Methane metabolism	173	9.95	33	2.42E-10	3.64E-08
	Pyruvate metabolism	113	6.5	14	0.00468	0.351
	Carbon fixation pathways in prokaryotes	97	5.58	12	0.00875	0.438
	Fatty acid degradation	25	1.44	5	0.0123	0.463
	Butanoate metabolism	97	5.58	11	0.0219	0.497
	Tryptophan metabolism	39	2.24	6	0.0222	0.497
	Drug metabolism—other enzymes	20	1.15	4	0.0248	0.497
	Lysine biosynthesis	41	2.36	6	0.0279	0.497
	Citrate cycle (TCA cycle)	54	3.11	7	0.0331	0.497
	Nitrogen metabolism	54	3.11	7	0.0331	0.497
	Arginine biosynthesis	45	2.59	6	0.0418	0.57
Module	Methanogenesis, CO2 = > methane	23	1.59	14	1.27E-11	4.31E-09
	F420 biosynthesis, archaea	5	0.346	5	1.46E-06	0.000164
	Acetyl-CoA pathway, CO2 = > acetyl-CoA	5	0.346	5	1.46E-06	0.000164
	Methanogenesis, acetate = > methane	19	1.31	8	1.64E-05	0.00138
	Lysine biosynthesis, acetyl-DAP pathway, aspartate = > lysine	9	0.622	4	0.00209	0.116
	Benzoyl-CoA degradation, benzoyl-CoA = > 3-hydroxypimeloyl-CoA	9	0.622	4	0.00209	0.116
	Dissimilatory sulfate reduction, sulfate = > H2S	5	0.346	3	0.00291	0.116
	Lysine biosynthesis, DAP aminotransferase pathway, aspartate = > lysine	10	0.692	4	0.00329	0.116
	Denitrification, nitrate = > nitrogen	10	0.692	4	0.00329	0.116
	Reductive acetyl-CoA pathway (Wood-Ljungdahl pathway)	16	1.11	5	0.00342	0.116
	Ornithine biosynthesis, glutamate = > ornithine	12	0.83	4	0.00696	0.214
	Lysine biosynthesis, succinyl-DAP pathway, aspartate = > lysine	13	0.899	4	0.00953	0.268
	Citrate cycle, second carbon oxidation, 2-oxoglutarate = > oxaloacetate	25	1.73	5	0.0252	0.548
	Lysine degradation, lysine = > saccharopine = > acetoacetyl-CoA	4	0.277	2	0.0259	0.548
	Phosphatidylethanolamine (PE) biosynthesis, PA = > PS = > PE	4	0.277	2	0.0259	0.548
	Fumarate reductase, prokaryotes	4	0.277	2	0.0259	0.548
	Benzoate degradation, cyclohexanecarboxylic acid = > pimeloyl-CoA	5	0.346	2	0.0413	0.821

Functional categories enriched with an FDR p < 0.05 are highlighted in bold

**Fig. 16:**
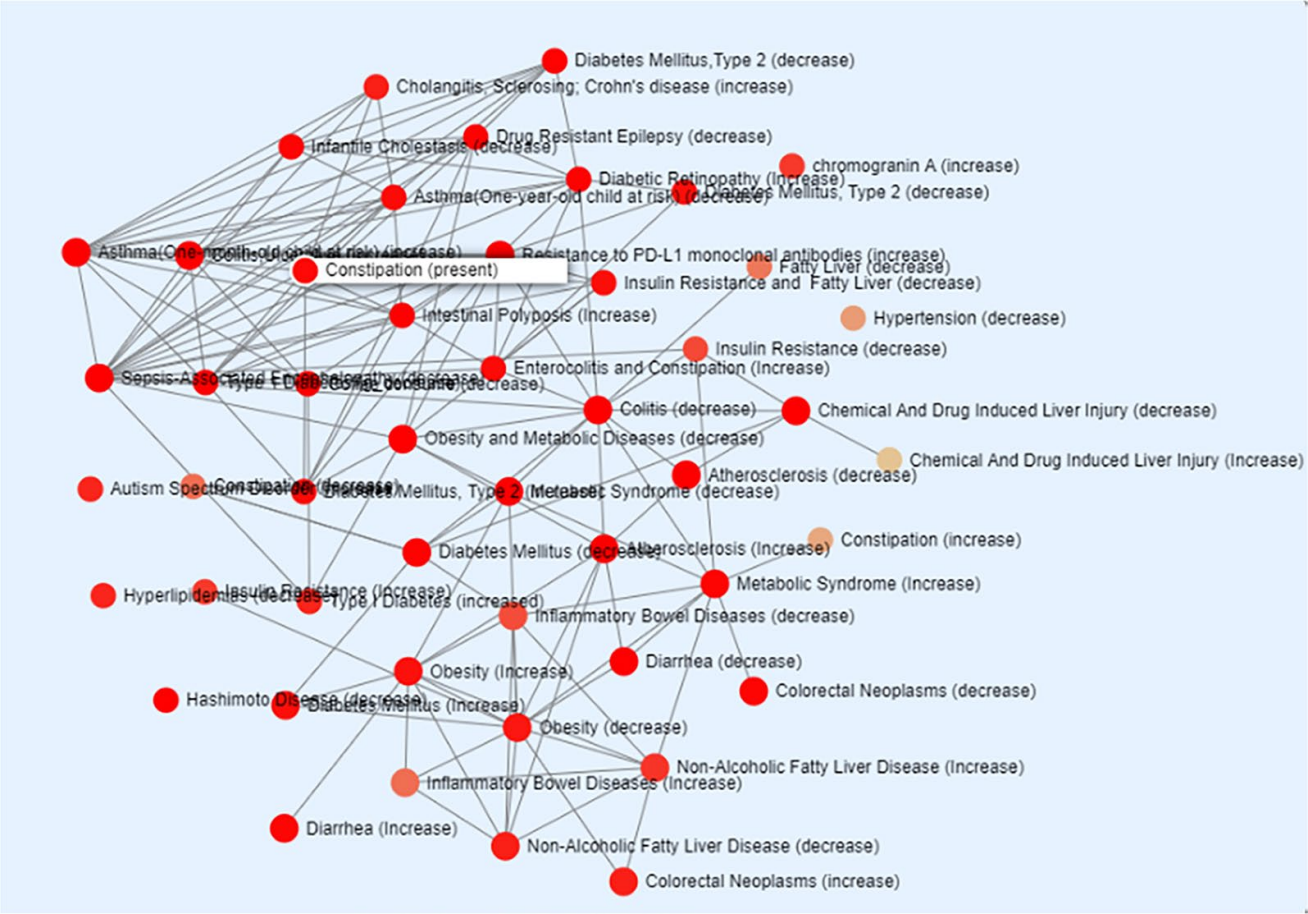
TSEA (intrinsic factor) of combined group taxa. Taxa (genus/species) with at least 10% abundance were considered for the taxa enrichment analysis

The TSEA enrichment analysis was performed using the taxa with at least 10% abundance. For the C group, “Colitis (decrease)” was among the significant (p = 1.81E-6) associations based on the host-intrinsic taxon set, with bacteria from *Lactobacillus*, *Bifidobacterium* and *Bacteroides* genera [26] (Fig. 16). Other groups were not considered for the enrichment analysis as very few taxa were found with 10% abundance.

## Discussion

IBD is a multifactorial disease, with a complex etiological factors contributing to its pathogenesis which involve an important role for the the gut microbiome [27, 28]. The current study examined the gut metagenome of UT healthy mice, and mice treated with DSS to induces a state of colonic inflammation plus treatment with vehicle (V), minocycline alone (M), methyl prednisolone alone (MP), or combination regimen of minocycline and methyl prednisolone (C).

The histological data indicated a decrease in the severity of colitis in the treatment with minocycolne or methyl prednisolone, while the combination regimen resulted in sunergistic and more robust decrease in the severity of colitis. Several taxa were found to be divergent in samples from mice with colitis compared to the UT or V treated groups. We observed a significant difference in the abundance profiles of certain bacteria in the treatment Vs. UT group. The microbiota from the mice treated with varios drug regimens displayed an overall reduction in the bacteria from the phyla *Firmicutes*. Several reports showed that this most abundant flora is decreased in IBD patients compared to healthy individuals [28–30], which is in aggrement with our findings in this report. Also, other flora such as *Actinobacteria* which is increased in a state of colinic inflammation was also found to be increased in our study when combared with samples taken from mice treated with various drug regiments. In a report by Shang and Liu, they found that microbes from *Muribaculaceae* and* Lachnospiraceae* genera to have a negative correlation with the development of colitis [31]. Interestingly, bacteria from *Muribaculaceae* along with *Bacteroides, Bifidobacterium, and Lactobacillus* were found to be predominant in the C group, and the bacteria from *Lachnospiraceae NK4A136 group* and *Helicobacter* were found to be predominant in M group. Bacteria from *Helicabacter* were found to be predominant in both MP and V groups. There is a known correlation between *Helicobacter* and UC, however, whether it is a causal relationship remains unclear [32]. The microbial flora from the *Weissella* genus found to be predominant in the UT group. A recent study suggested that the bacteria from this genus may have antimicrobial and probiotic effects [33].

The abundance of the bacteria from the genus *Dorea* was found to be significantly higher in C compared to V and UT groups. There is a known association of this bacteria with UC and CD pathogenesis [34]. Similarily, the abundance of *Monoglobus* genera was found to be higher in treatment groups compared to V and UT groups. A recent study by Zhu et al., identified a decreased abundance of this bacteria in UC pateints [35], corroborating with the current findings. On the contrary, microbes from *Rikenellaceae_RC9_gut_group*, a known inducer of colitis [36] were found to be significantly low in C group. The bacteria from this genera is also known to be associated with the consumption of high-fat diet [37].

At the genus level, *Eubacterium siraeum group*, *Clostridia vadin BB60 group*, *Erysipelatoclostridium* and *Anaeroplasma* have a significant increase in their abundance in the samples from the V group compared to the C and UT groups. *Eubacterium siraeum* is one of the four well-characterized microbial-serpin contributing bacteria and is known for the host-bacterium crosstalk [38, 39]. Serine protease inhibitors (serpins) are well-known for their anti-inflammatory properties and shown to play an important role in IBD [39]. *Anaeroplasma* is another group of bacteria negatively associated with inflammation [40] that were found to be increased significantly in V group samples compared to UT and drug-treated (M, MP, and C) samples. An increased abundance was observed in *Erysipelatoclostridium* among the vehicle-treated (V) samples. A study by Zhibing et al., found a dramatic increase in the abundance of this bacteria in Crohn’s disease patients [41]. The bacterial species (*Erysipelatoclostridium ramosum DSM 1402*) from this genera were also characterized for their metabolic potential in ulcerative colitis and Crohn’s disease [42]. The current study also identified microbes from the *Clostridia vadin BB60 group* to be significantly altered in DSS/vehicle-treated samples. These bacteria are known to be associated with diet and human health [43–45].

At the species level, *Helicobacter mastomyrinus* and *Massiliomicrobiota timonensis* were significantly increased in V group compared to UT as well as M and C groups. A study by Kathryn et al., identified that *Helicobacter mastomyrinus* can cause severe ulcerative typhlocolitis in telomerase deficient mice [46]. Whereas, *Massiliomicrobiota timonensis* species is hardly reported to be associated with colitis. However, a recent study identified significant alterations in the abundance of this bacteria in ultramarathon runners [47]. Their involvement in affecting the host immune function needs to be further explored.

Species of the *Lactobacillus* are used as probiotics as well as most widely used in UC therapy [48, 49]. Intrestingly, in the current study, *Lactobacillus intestinalis* was found to increase significantly with C regimen, indicating it as a possible target for UC therapy. The TSEA analysis also identified “Colitis (decrease)” as asignificant association with the taxa obtained form the current analysis that included *Lactobacillus* [26]. Methane positivity is well associated with IBD pathogenesis [50], and the functional analysis of the significantly different KEGG IDs across groups revealed methane metabolism as the enriched pathway.

The findings from this study suggests that the treatment with a combination of M and MP can alter the gut microbiota and contributes to the reduction of severity of colitis and may serve as a promising therapeutic option.

### Supplementary Information

**Additional file 1: Figure S1.** Core microbiomeassociated with combined group. **Figure S2.** Core microbiomeat the genus level associated with Minocycline group. **Figure S3.** Core microbiomeat the genus level associated with Methyl prednisolone group. **Figure S4.** Core microbiomeat the genus level associated with Vehicle group. **Figure S5.** Core microbiomeat the genus level associated with Untreated group.

## Data Availability

The datasets used and/or analysed during the current study are available from the corresponding author on reasonable request.

## References

[1] Ng SC (2017). Worldwide incidence and prevalence of inflammatory bowel disease in the 21st century: a systematic review of population-based studies. Lancet.

[2] Boyapati R, Satsangi J, Ho GT (2015). Pathogenesis of Crohn's disease. F1000Prime Rep.

[3] Xavier RJ, Podolsky DK (2007). Unravelling the pathogenesis of inflammatory bowel disease. Nature.

[4] de Lange KM, Barrett JC (2015). Understanding inflammatory bowel disease via immunogenetics. J Autoimmun.

[5] Hanauer SB (2006). Inflammatory bowel disease: epidemiology, pathogenesis, and therapeutic opportunities. Inflamm Bowel Dis.

[6] Koloski NA, Bret L, Radford-Smith G (2008). Hygiene hypothesis in inflammatory bowel disease: a critical review of the literature. World J Gastroenterol.

[7] Zatorski H, Nakov R (2020). Faecal microbiota transplantation in inflammatory bowel disease: current concepts and future challenges. Curr Drug Targets.

[8] Wilson ID, Nicholson JK (2009). The role of gut microbiota in drug response. Curr Pharm Des.

[9] Sellon RK (1998). Resident enteric bacteria are necessary for development of spontaneous colitis and immune system activation in interleukin-10-deficient mice. Infect Immun.

[10] Taurog JD (1994). The germfree state prevents development of gut and joint inflammatory disease in HLA-B27 transgenic rats. J Exp Med.

[11] Packey CD, Sartor RB (2009). Commensal bacteria, traditional and opportunistic pathogens, dysbiosis and bacterial killing in inflammatory bowel diseases. Curr Opin Infect Dis.

[12] Perencevich M, Burakoff R (2006). Use of antibiotics in the treatment of inflammatory bowel disease. Inflamm Bowel Dis.

[13] Khajah MA, Fateel MM, Ananthalakshmi KV, Luqmani YA (2016). Anti-Inflammatory action of angiotensin 1–7 in experimental colitis. PLoS One.

[14] Wirtz S, Neufert C, Weigmann B, Neurath MF (2007). Chemically induced mouse models of intestinal inflammation. Nat Protoc.

[15] Park H, Yeo S, Kang S, Huh CS (2021). Longitudinal microbiome analysis in a dextran sulfate sodium-induced colitis mouse model. Microorganisms.

[16] Kozik AJ, Nakatsu CH, Chun H, Jones-Hall YL (2019). Comparison of the fecal, cecal, and mucus microbiome in male and female mice after TNBS-induced colitis. PLoS One.

[17] Dinov ID (2008). iTools: a framework for classification, categorization and integration of computational biology resources. PLoS ONE.

[18] Kechin A, Boyarskikh U, Kel A, Filipenko M (2017). cutPrimers: a new tool for accurate cutting of primers from reads of targeted next generation sequencing. J Comput Biol.

[19] Hall M, Beiko RG (2018). 16S rRNA gene analysis with QIIME2. Methods Mol Biol.

[20] Quast C (2013). The SILVA ribosomal RNA gene database project: improved data processing and web-based tools. Nucleic Acids Res.

[21] Katoh K, Standley DM (2013). MAFFT multiple sequence alignment software version 7: improvements in performance and usability. Mol Biol Evol.

[22] Price MN, Dehal PS, Arkin AP (2010). FastTree 2–approximately maximum-likelihood trees for large alignments. PLoS ONE.

[23] Dhariwal A (2017). Microbiome analyst: a web-based tool for comprehensive statistical, visual and meta-analysis of microbiome data. Nucleic Acids Res.

[24] Aßhauer KP, Wemheuer B, Daniel R, Meinicke P (2015). Tax4Fun: predicting functional profiles from metagenomic 16S rRNA data. Bioinformatics.

[25] Goeman JJ, van de Geer SA, de Kort F, van Houwelingen HC (2004). A global test for groups of genes: testing association with a clinical outcome. Bioinformatics.

[26] Liu S (2016). The host shapes the gut microbiota via fecal MicroRNA. Cell Host Microbe.

[27] Ni J, Wu GD, Albenberg L, Tomov VT (2017). Gut microbiota and IBD: causation or correlation?. Nat Rev Gastroenterol Hepatol.

[28] Nishida A (2018). Gut microbiota in the pathogenesis of inflammatory bowel disease. Clin J Gastroenterol.

[29] Scanlan PD, Shanahan F, O'Mahony C, Marchesi JR (2006). Culture-independent analyses of temporal variation of the dominant fecal microbiota and targeted bacterial subgroups in Crohn's disease. J Clin Microbiol.

[30] Ott SJ (2004). Reduction in diversity of the colonic mucosa associated bacterial microflora in patients with active inflammatory bowel disease. Gut.

[31] Shang L (2021). Core altered microorganisms in colitis mouse model: a comprehensive time-point and fecal microbiota transplantation analysis. Antibiotics (Basel).

[32] Mansour L (2018). Helicobacter pylori may be an initiating factor in newly diagnosed ulcerative colitis patients: A pilot study. World J Clin Cases.

[33] Ahmed S (2022). The weissella genus: clinically treatable bacteria with antimicrobial/probiotic effects on inflammation and cancer. Microorganisms.

[34] Seishima J (2019). Gut-derived Enterococcus faecium from ulcerative colitis patients promotes colitis in a genetically susceptible mouse host. Genome Biol.

[35] Zhu S (2022). Composition and diverse differences of intestinal microbiota in ulcerative colitis patients. Front Cell Infect Microbiol.

[36] Wang JL (2022). Differential analysis of intestinal microbiota and metabolites in mice with dextran sulfate sodium-induced colitis. World J Gastroenterol.

[37] Zhou L (2018). Improved glucose and lipid metabolism in the early life of female offspring by maternal dietary genistein is associated with alterations in the gut microbiota. Front Endocrinol.

[38] Mkaouar H (2016). Siropins, novel serine protease inhibitors from gut microbiota acting on human proteases involved in inflammatory bowel diseases. Microb Cell Fact.

[39] Mkaouar H (2021). Gut serpinome: emerging evidence in IBD. Int J Mol Sci.

[40] Monk JM (2016). Diets enriched with cranberry beans alter the microbiota and mitigate colitis severity and associated inflammation. J Nutr Biochem.

[41] Qiu Z (2017). Targeted metagenome based analyses show gut microbial diversity of inflammatory bowel disease patients. Indian J Microbiol.

[42] Sankarasubramanian J, Ahmad R, Avuthu N, Singh AB, Guda C (2020). Gut microbiota and metabolic specificity in ulcerative colitis and Crohn's Disease. Front Med.

[43] Deng L (2023). Colonization with ubiquitous protist Blastocystis ST1 ameliorates DSS-induced colitis and promotes beneficial microbiota and immune outcomes. NPJ Biofilms Microbiomes.

[44] Yu J (2023). Disruption of the intestinal mucosal barrier induced by high fructose and restraint stress is regulated by the intestinal microbiota and microbiota metabolites. Microbiol Spectr.

[45] Tindall AM, McLimans CJ, Petersen KS, Kris-Etherton PM, Lamendella R (2020). Walnuts and vegetable oils containing oleic acid differentially affect the gut microbiota and associations with cardiovascular risk factors: follow-up of a randomized, controlled, feeding trial in adults at risk for cardiovascular disease. J Nutr.

[46] Eaton KA, Opp JS, Gray BM, Bergin IL, Young VB (2011). Ulcerative typhlocolitis associated with Helicobacter mastomyrinus in telomerase-deficient mice. Vet Pathol.

[47] Sato M, Suzuki Y (2022). Alterations in intestinal microbiota in ultramarathon runners. Sci Rep.

[48] Štofilová J (2022). Probiotic-based intervention in the treatment of ulcerative colitis: conventional and new approaches. Biomedicines.

[49] Zocco MA (2006). Efficacy of lactobacillus GG in maintaining remission of ulcerative colitis. Aliment Pharmacol Ther.

[50] Gandhi A (2021). Methane positive small intestinal bacterial overgrowth in inflammatory bowel disease and irritable bowel syndrome: a systematic review and meta-analysis. Gut Microbes.

