# Supplementary material for: Effect of minocycline, methyl prednisolone, or combination treatment on the colonic bacterial population in a state of colonic inflammation using the murine dextran sulfate sodium model
Source: Microb Cell Fact. 2023 Nov 10;22:232. doi: 10.1186/s12934-023-02242-8 (PMC10636938; doi:10.1186/s12934-023-02242-8)
Supplement: Supplementary file 1 — Additional file 1: Figure S1. Core microbiomeassociated with combined group. Figure S2. Core microbiomeat the genus level associated with Minocycline group. Figure S3. Core microbiomeat the genus level associated with Methyl prednisolone group. Figure S4. Core microbiomeat the genus level associated with Vehicle group. Figure S5. Core microbiomeat the genus level associated with Untreated group. [file 12934_2023_2242_MOESM1_ESM.pdf]

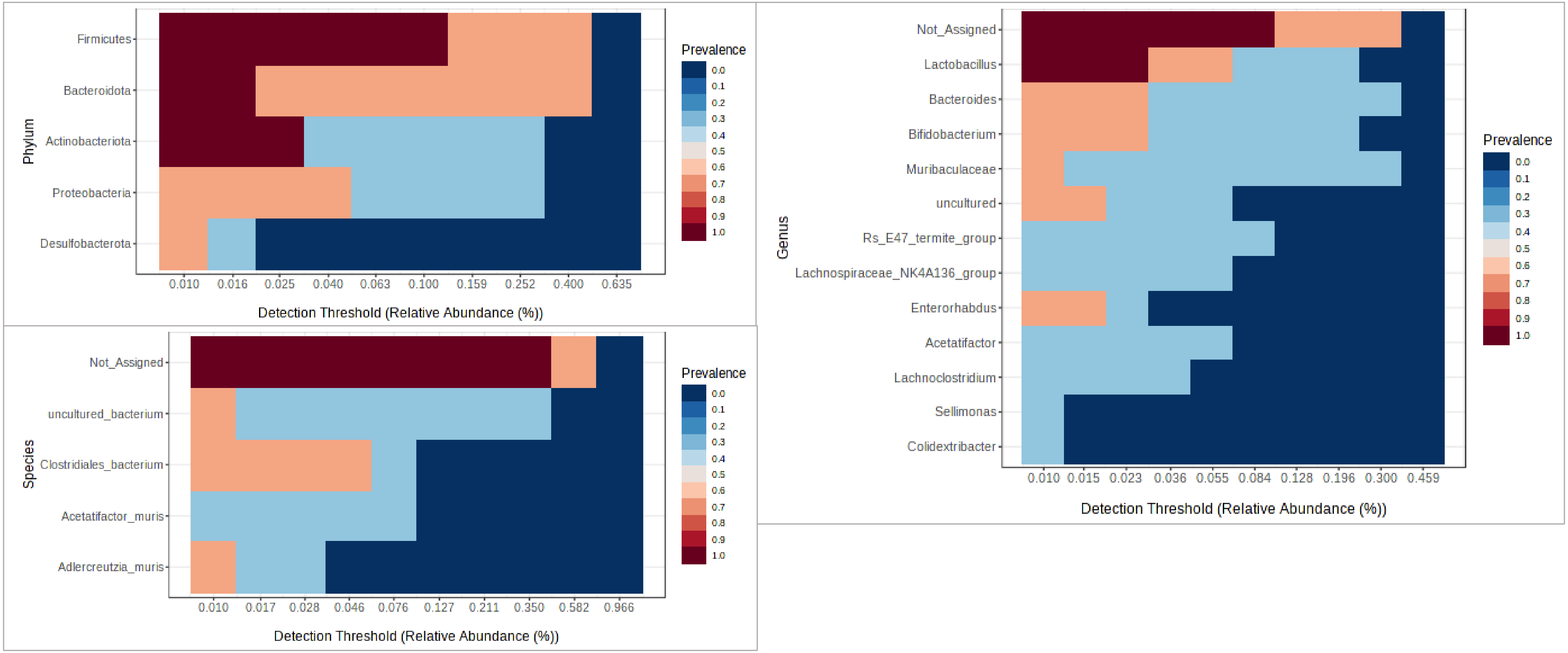

Figure S1: Core microbiome associated with combined group

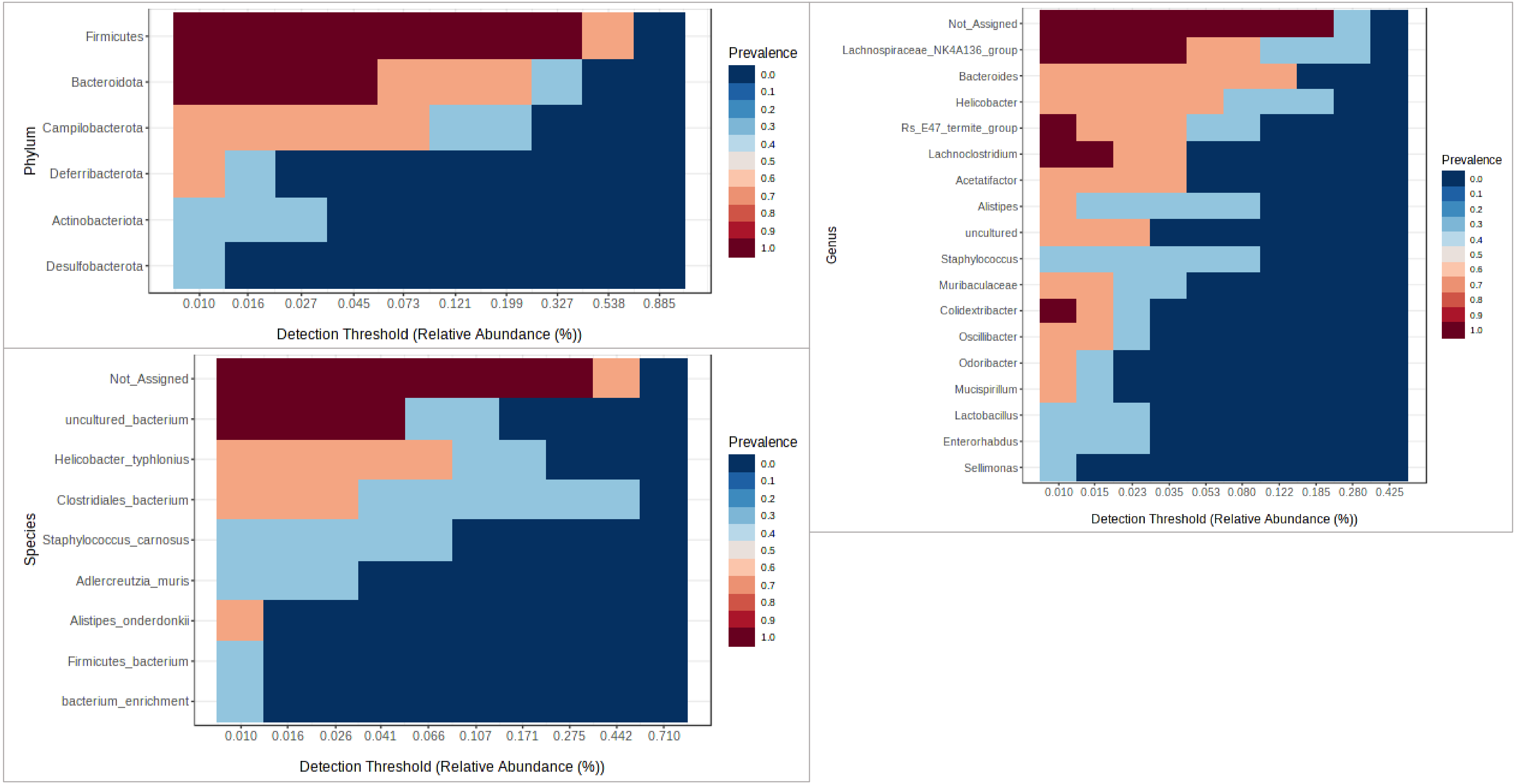

Figure S2: Core microbiome at the genus level associated with Minocycline group

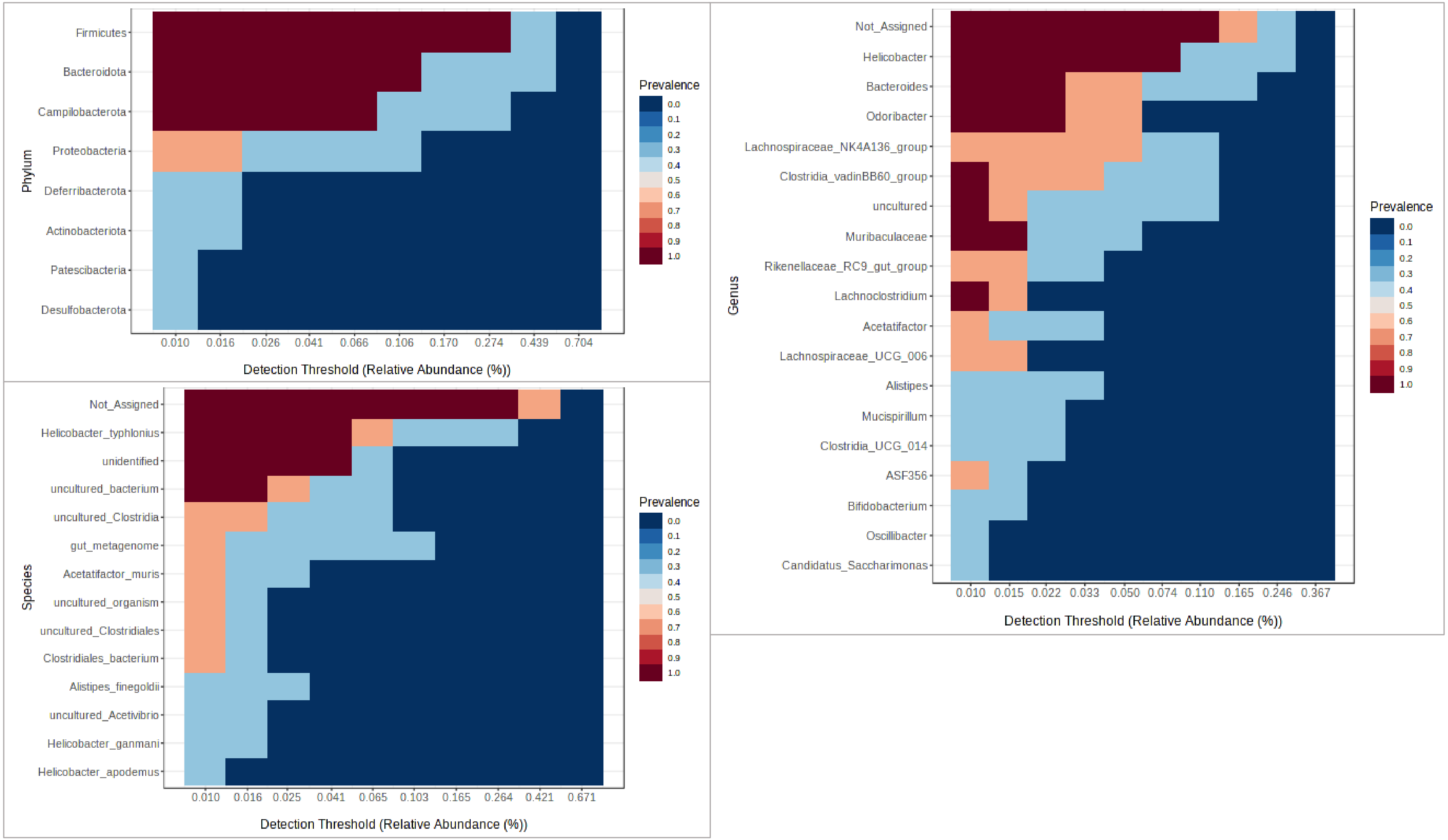

Figure S3: Core microbiome at the genus level associated with Methyl prednisolone group

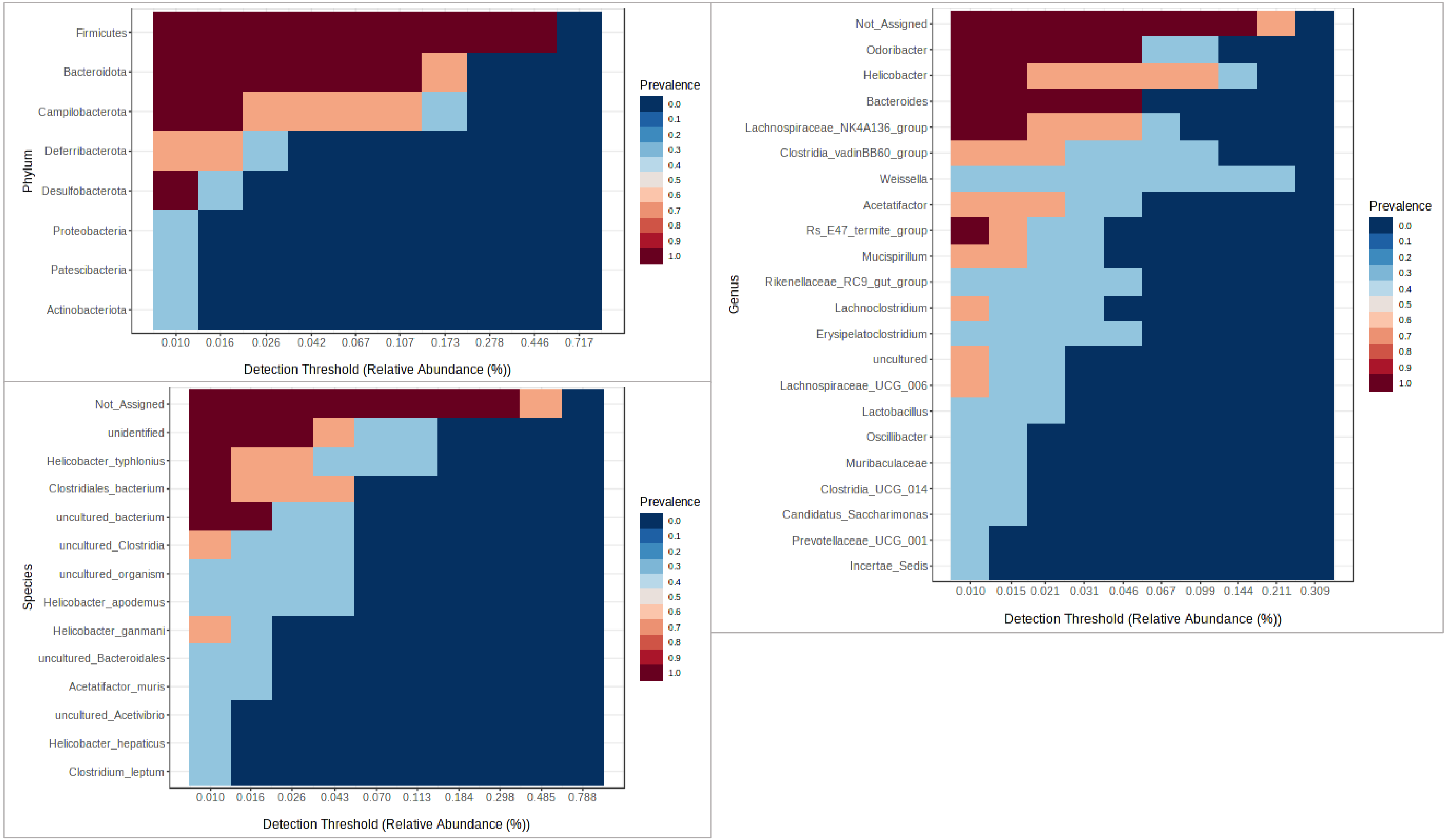

Figure S4: Core microbiome at the genus level associated with Vehicle group

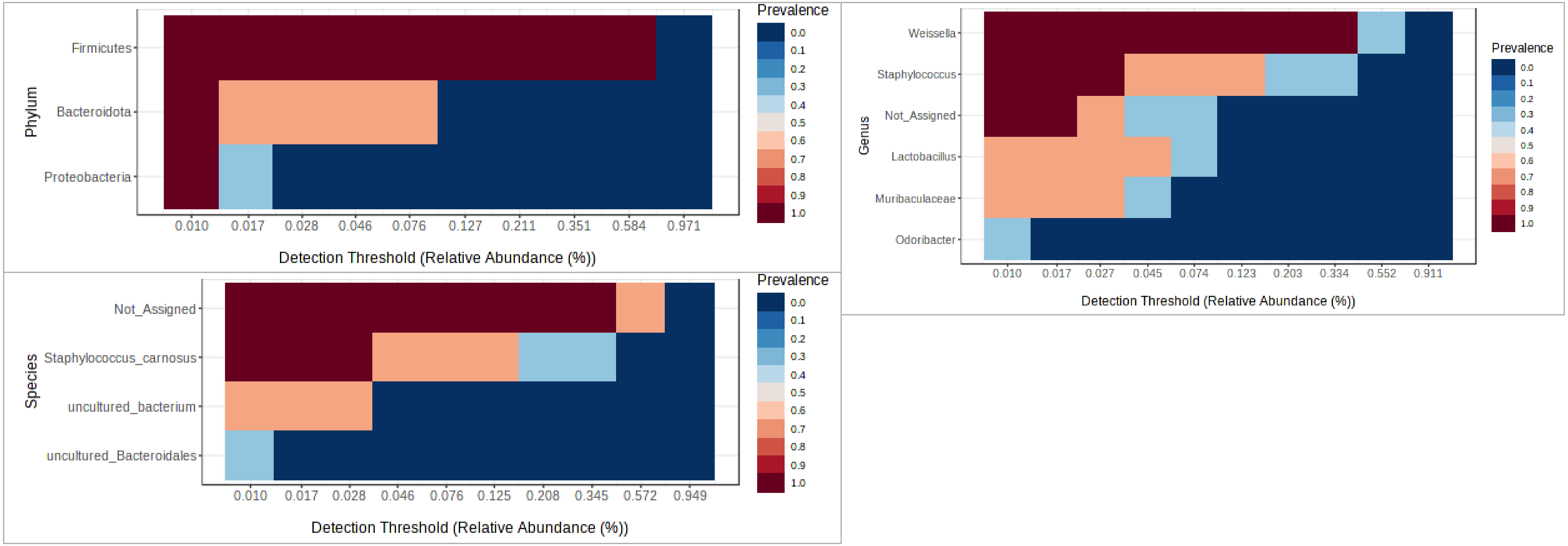

Figure S5: Core microbiome at the genus level associated with Untreated group
